# Development of scoring-assisted generative exploration (SAGE) and its application to dual inhibitor design for acetylcholinesterase and monoamine oxidase B

**DOI:** 10.1186/s13321-024-00845-w

**Published:** 2024-05-24

**Authors:** Hocheol Lim

**Affiliations:** Bioinformatics and Molecular Design Research Center (BMDRC), Incheon, Republic of Korea

**Keywords:** Drug discovery, De novo molecular design, Fine-tuning, Quantitative structure–activity relationship, Dual inhibitor design

## Abstract

**Abstract:**

De novo molecular design is the process of searching chemical space for drug-like molecules with desired properties, and deep learning has been recognized as a promising solution. In this study, I developed an effective computational method called Scoring-Assisted Generative Exploration (SAGE) to enhance chemical diversity and property optimization through virtual synthesis simulation, the generation of bridged bicyclic rings, and multiple scoring models for drug-likeness. In six protein targets, SAGE generated molecules with high scores within reasonable numbers of steps by optimizing target specificity without a constraint and even with multiple constraints such as synthetic accessibility, solubility, and metabolic stability. Furthermore, I suggested a top-ranked molecule with SAGE as dual inhibitors of acetylcholinesterase and monoamine oxidase B through multiple desired property optimization. Therefore, SAGE can generate molecules with desired properties by optimizing multiple properties simultaneously, indicating the importance of de novo design strategies in the future of drug discovery and development.

**Scientific contribution:**

The scientific contribution of this study lies in the development of the Scoring-Assisted Generative Exploration (SAGE) method, a novel computational approach that significantly enhances de novo molecular design. SAGE uniquely integrates virtual synthesis simulation, the generation of complex bridged bicyclic rings, and multiple scoring models to optimize drug-like properties comprehensively. By efficiently generating molecules that meet a broad spectrum of pharmacological criteria—including target specificity, synthetic accessibility, solubility, and metabolic stability—within a reasonable number of steps, SAGE represents a substantial advancement over traditional methods. Additionally, the application of SAGE to discover dual inhibitors for acetylcholinesterase and monoamine oxidase B not only demonstrates its potential to streamline and enhance the drug development process but also highlights its capacity to create more effective and precisely targeted therapies. This study emphasizes the critical and evolving role of de novo design strategies in reshaping the future of drug discovery and development, providing promising avenues for innovative therapeutic discoveries.

**Supplementary Information:**

The online version contains supplementary material available at 10.1186/s13321-024-00845-w.

## Introduction

Discovering new molecules with the desired properties is a key aspect of drug discovery, but the task of finding these molecules is challenging because of the massive size of the chemical space. While virtual screening is an efficient method for rapidly identifying compounds from existing commercial databases, it’s gradually becoming more challenging to find molecules that both satisfy all desired properties and avoid patent infringement. To overcome this challenge, de novo molecular design has been developed as a solution [1–3], which aims to create new molecules with the desired properties from scratch. Generative deep learning has revolutionized the field of de novo molecular design by enabling direct learning from input data without relying on human-made rules. This approach has shown great success in the field of de novo molecular design by effectively exploring uncharted chemical space for drugs and creating new molecules with specific properties [4–6]. For example, genetic expert-guided learning (GEGL) demonstrated impressive performance in several de novo molecular design tasks and added genetic algorithms (GA) to generative deep learning through the domain-specific genetic operator, which allows for effective exploration of the chemical space [7]. This approach has the potential to revolutionize drug discovery by offering a more effective method of identifying potential drug candidates, but it is necessary to validate the drug-likeness of these newly designed molecules.

Drug-likeness is a crucial element in drug discovery, which helps to increase the success rate of clinical trials, reduce costs, and filter out compounds with a high likelihood of failure. Computational filters have been developed to distinguish between drug-like and non-drug-like molecules, such as Lipinski’s rules [8]. However, relying solely on a single drug-likeness property has limitations in drug discovery, as the definition of drug-likeness has expanded to include various molecular properties that may not necessarily be related to drug efficacy or safety. Therefore, it is necessary to separately consider pharmacodynamic and pharmacokinetic properties in the drug discovery process for effective drugs. Pharmacodynamic properties relate to a drug molecule's ability to interact specifically with a biological target without causing off-target effects. Pharmacokinetic properties determine if a drug molecule will reach its target protein and persist in the bloodstream, and this includes processes such as absorption, distribution, metabolism, and excretion (ADME), while toxicity refers to adverse or harmful drug effects, and its examination is crucial for identifying potential risks and reducing negative side effects. Improving drug-likeness requires optimizing multiple factors such as target specificity and ADME/T together, and this is essential for discovering effective drugs.

Targeting a single protein has been successful in managing many diseases, but complex diseases require alternative approaches like combination therapy and multi-target drugs. Dual-action drugs have two distinct desired effects at a single effective dose from two separate modes of action, making them versatile for treating a variety of diseases. For instance, acetylcholinesterase (AChE) inhibitors increase cholinergic levels in the brain to treat Alzheimer’s disease (AD), by enhancing the cholinergic levels in the brain [9], while monoamine oxidase (MAO) inhibitors reduce oxidative damage and have the potential for treating AD [10]. AChE/MAO dual inhibitors are believed to be more effective in treating AD, and ladostigil showed a neuroprotective ability and stimulated the processing of amyloid precursor protein (APP) alpha through AChE/MAO dual inhibition [10, 11]. However, computationally designing AChE/MAO dual inhibitors is a challenging task due to the complex balance of target specificity.

Quantitative structure–activity/property relationship (QSAR/QSPR) methods have been widely used for predicting target specificity and ADME/T properties by identifying molecular features in known active and inactive ligands. While QSAR models can help eliminate undesirable compounds during drug design and provide feedback for lead optimization, they are not able to create or generate new molecules with desired properties. Therefore, QSAR models should be combined with other computational methods to generate new molecules with desired properties.

In this study, I devised an effective computational methodology named Scoring-Assisted Generative Exploration (SAGE) by integrating the GEGL framework and multiple QSAR models. My SAGE expanded the GEGL for practical use by enabling virtual synthesis simulation, generating bridged bicyclic rings for greater chemical diversity, and adding multiple scoring models for drug-likeness. Firstly, I performed pretraining of SAGE on various datasets to create a model capable of the most diverse compounds. Secondly, I carried out a benchmark to generate compounds for finding the bridged bicyclic ring structures based on the presence or absence of chemical diversification. Thirdly, I evaluated the chemical design ability of SAGE in generating compounds with desired properties by optimizing drug-likeness in six protein targets using six QSAR models for target specificity and 11 QSPR models for ADME/T properties. Lastly, I performed a task of identifying a dual inhibitor of AChE and MAO type B (MAOB) using two QSAR properties for target specificity and 12 QSPR models for ADME/T properties. My results showed that SAGE can effectively explore chemical space and optimize multiple properties using various scoring models, making it useful for discovering molecules with desired properties at an early stage of drug discovery.

## Methods

### Scoring-assisted generative exploration (SAGE)

Scoring-Assisted Generative Exploration (SAGE) is an effective framework for generating high-scoring molecules with deep neural networks (DNN), chemical diversification operators, and various scoring models for desired objectives. The DNN in SAGE is pre-trained with chemical datasets and based on long-short-term memory (LSTM) networks [12]. Molecules are represented as a sequence of characters in the simplified molecular-input line-entry system (SMILES) format [13]. Chemical diversification operators in SAGE consist of mutate, crossover, and virtual synthesis operators. The mutate operator makes various chemical modifications to the molecules at the atom level, such as appending atoms, inserting atoms, changing bond orders in covalent bonds, adding ring bonds, deleting ring bonds, and forming a bridge bicyclic ring in a ring substructure. The crossover operator randomly breaks a pair of parent molecules into fragments and combines two fragments to create a new molecule at the functional group level, where a fragment can be attached to the bridgehead atoms in bridged bicyclic rings. The virtual synthesis operator is based on a virtual assembly employed in the design of innovative new chemical entities generated by optimization strategies (DINGOS) [14] at the molecule level. The DINGOS algorithm consists of four key steps, including the generation of the molecular building block library, choosing a subset of the closest molecules to the original structure, construction of optimal intermediates and products, and repeating the process until a termination criterion is met. As a result, it allows for the generation of novel compounds through scaffold hopping based on ligand similarity and feasibility at the molecule level. After chemical generation and diversification, molecules are ranked based on their scores, and a fixed number of top-ranked molecules are selected for fine-tuning of the DNN through a storage buffer in every step. It is worth noting that the top-ranked molecules in the storage buffer are maintained throughout the entire process, regardless of the step.

The compounds used for pre-training the SAGE models were categorized into three groups, namely synthetic compounds, natural products, and bioactive compounds, and are summarized in Table 1. Two groups of synthetic compounds were obtained from the ZINC Clean Leads [15, 16], and 11 commercially available vendors (BIONET, Chembridge, ChemDiv, Enamine, IBS, LifeChemical, Maybridge, MolPort, Specs, TargetMol, and VitasM). Natural products were collected from ZINC20 [17], while bioactive compounds were collected from ChEMBL24 [18]. The compounds were randomly partitioned into training, validation, and test sets with proportions of 0.882, 0.098, and 0.02, respectively.

**Table 1:** Summary of datasets used in this study

Class	Task	Abbreviation	Unit	All set (active/inactive)	Training set	Test set
Pre-train	ZINC Clean Leads	ZINC	–	249,456	244,456	5000
	Commercial Vendors	Synthetics		17,134,091	16,791,409	342,682
	Natural Products	ZINC-NP		234,997	230,297	4700
	Bioactives	ChEMBL24		369,860	362,463	7397
Target specificity	Acetylcholinesterase	AChE	Binary	940 (453/487)	752	188
	Cyclooxygenase-2	COX-2		879 (435/444)	703	176
	Protein kinase C beta	PKCB		288 (135/153)	230	58
	Fibroblast growth factor receptor 1	FGFR1		285 (139/146)	228	57
	Protein-tyrosine phosphatase 1B	PTP1B		283 (130/153)	226	57
	Monoamine oxidase B	MAOB		251 (122/129)	200	51
Absorption	Caco-2 membrane permeability	Caco2	cm/s	910	728	182
	Human intestinal absorption	HIA	Binary	578 (500/78)	461	117
	P-glycoprotein inhibition	Pgp		1218 (650/568)	973	245
Distribution	Human plasma protein binding rate	PPBR	%	2790	2231	559
	Blood–brain barrier permeability	BBB	Binary	2030 (1551/479)	1624	406
Metabolism	CYP-P450 inhibition (CYP2D6)	CYP2D6	Binary	13,130 (2514/10,616)	10,504	2626
	CYP-P450 inhibition (CYP3A4)	CYP3A4		12,328 (5110/7218)	9861	2467
	CYP-P450 inhibition (CYP2C9)	CYP2C9		12,092 (4045/8047)	9673	2419
Toxicity	Lethal dose 50	LD50	log(1/mol/kg)	7385	5907	1478
	Human ether-à-go-go	hERG	Binary	655 (451/204)	523	132
	Mutagenicity	AMES		7278 (3974/3304)	5821	1457
	Drug-induced liver injury	DILI		475 (236/239)	379	96

The DNN in SAGE for pretraining comprises a 3-layered LSTM with 1024 hidden units and a dropout probability of 0.2. Optimization was performed using the Adam optimizer [19], with a learning rate of 0.001 and a batch size of 1024. The DNN was pre-trained for 300 epochs on four datasets (ChEMBL24, Synthetics, ZINC, and ZINC-NP) and the final model weights were selected based on the minimum average loss on the validation and test sets. The generated molecules were evaluated for validity, uniqueness, novelty, and internal diversity. Validity assesses how well the DNN incorporates chemically reasonable constraints and grammar in SMILES, such as maintaining proper valence. Uniqueness measures how well the DNN avoids generating only a few typical molecules, while Unique_1k is the uniqueness obtained by examining the first 1000 valid molecules in the generated set. Novelty calculates the percentage of generated molecules that do not exist in the training set. IntDiv_1 and IntDiv_2 measure the chemical diversity within the generated molecule sets, with higher scores indicating greater diversity.

SAGE adopts an iterative fine-tuning approach, wherein each iteration leverages the principles of fine-tuning and reuses the parameters in the pretraining phase. This strategy allows the model to retain the knowledge gained through pretraining while fine-tuning it on the target task, resulting in improved performance and convergence. During each iteration of SAGE, the DNN generates 8192 molecules, which undergo filtering to remove invalid SMILES or non-drug-like molecules through Muegges drug-like filters. The remaining molecules are then evaluated using scoring models that are suitable for each task objective, and the top 1024 molecules are saved in the storage buffer. The crossover operator is then applied to at most 8192 pairs of molecules randomly selected from the storage buffer. If the crossover operator cannot be applied due to overly simplistic molecules, the operation is skipped with a probability of 0.01. Upon a successful crossover operation, the mutate (M), virtual synthesis (V), and bridged bicyclic (B) operators are applied according to pre-defined probabilities (M100, M75/V20/B05, M50/V45/B05, and M25/V70/B05). If a new molecule is not generated from the parent pair, the process can be repeated up to 10 to generate a new compound. The invalid or non-drug-like ones are filtered out, and the top 1024 molecules are saved in the storage buffer. The duplicate-removed storage buffer, which can have at most 1024, is used for fine-tuning the DNN. Optimization for the fine-tuning was performed using the Adam optimizer, with a learning rate of 0.001 and a batch size of 256 for 8 epochs. For benchmarking purposes, I conducted 100 iterations of SAGE for the GuacaMol and bridged bicyclic ring tasks and 50 iterations for inhibitor design tasks, with a maximum SMILES length of 100. To establish baseline models (SMILES LSTM and SMILES GA), I introduced specific modifications to SAGE. The reward parameter was fixed at a minimal value of 0.01, and I refrained from proceeding with the DNN training under these conditions. This approach effectively disables the ranking-based fine-tuning mechanism, a core component of SAGE, allowing us to isolate and analyze the algorithm's fundamental capabilities. The SMILES LSTM and SMILES GA models generated 16,384 molecules in the GuacaMol benchmark, while the SAGE in inhibitor design tasks generated 8,192 molecules through SMILES LSTM and 8192 molecules through SMILES GA.

### Goal-directed benchmarks

Six goal-directed benchmarks for model performance evaluation were derived from the GuacaMol [20]. Rediscovery tasks involve the rediscovery of specific target compounds, namely Celecoxib, Troglitazone, Thiothixene, and all bridged bicyclic compounds. Meanwhile, similarity tasks aimed to generate molecules closely resembling specific target compounds, such as Aripiprazole, Aluterol, Mestranol, and all bridged bicyclic compounds. For each of these target compounds, I selected the top 100 generated molecules that exhibited a similarity score above a 0.75 threshold. In the rediscovery tasks of the GuacaMol and bridged bicyclic benchmarks, the similarity between each target compound and the top 1 generated molecule was assessed using the ECFP4 molecular fingerprint. The similarities between the respective molecules and Aripiprazole, Aluterol, Mestranol, and all bridged bicyclic compounds were measured using distinct molecular fingerprints: ECFP4 for Aripiprazole and all bridged bicyclic compounds, FCFP4 for Aluterol, and AP for Mestranol. Isomer tasks revolve around the creation of molecules that align with a given molecular formula, assessing the overfitting problem of only producing molecules with a simple pattern.

Multiple property optimization (MPO) tasks involve the modification of known drug molecules, such as Fexofenadine, Ranolazine, Perindopril, Amlodipine, Sitagliptin, and Zaleplon, for structural or physicochemical properties, where I selected the top 100 generated molecules and compared the multiple properties. In the Fexofenadine MPO task, I employed the geometric mean of AP fingerprint-based similarity, a LogP target of 4, and a topological polar surface area (TPSA) with a target of 90. For the Ranolazine MPO task, the geometric mean of AP fingerprint-based similarity, a LogP target of 7, a TPSA target of 95, and the presence of one fluorine atom were used as the optimization criteria. In the case of Perindopril MPO, I utilized the geometric mean of ECFP4 fingerprint-based similarity and the number of two aromatic rings. The Amlodipine MPO task involved using the geometric mean of ECFP4 fingerprint-based similarity and the presence of two carbon rings. Finally, the Sitagliptin MPO task included an ECFP4 fingerprint-based similarity, a LogP of 2, a TPSA of 77, and a simultaneous isomer task targeting the C_16_H_15_F_6_N_5_O molecular formula.

The Valsartan SMARTS task focuses on molecules that manifest SMARTS pattern associated with valsartan, and that have similar physicochemical properties with the sitagliptin. Scaffold- and decorator hopping tasks endeavor to maximize the congruity with SMILES string, either preserving or excluding particular SMARTS patterns. It can maintain specific substituents and modify the scaffold of a compound, while it can maintain a consistent scaffold and alter the substitution pattern. The scaffold and decorator hopping tasks in the GuacaMol used one SMARTS pattern for scaffold (‘[#7]-c1n[c;h1]nc2[c;h1]c(-[#8])[c;h0][c;h1]c12’) and three SMARTS patterns for decoration (‘CS([#6])(= O) = O’, ‘[#7]-c1ccc2ncsc2c1’, and ‘[#6]-[#6]-[#6]-[#8]-[#6] ~ [#6] ~ [#6] ~ [#6] ~ [#6]-[#7]-c1ccc2ncsc2c1’). To evaluate the ability to generate bridged bicyclic ring structures, I adopted four goal-directed benchmarks named rediscovery and similarity tasks. All similarities of the molecules with bridged bicyclic rings were measured with the ECFP4.

### Chemical filters and score definition

The development of a precisely defined scoring function was essential for the SAGE model’s effectiveness in generating chemicals with targeted properties. An inadequately defined scoring function could lead to suboptimal outcomes, diverging from intended objectives. To align the scoring functions with the desired molecular properties, I focused on five key factors: simple drug-likeness, target specificity, synthetic accessibility, solubility, and ADME/T properties.

Initially, my SAGE model involved implementing several chemical filters based on simple rule-based drug-likeness. These filters categorize molecules as potential drugs or non-drugs, drawing upon their similarity to known drugs in the feature space. To provide a rough estimate of a molecule’s drug-likeness, I employed the Muegge filter [21]. This filter disqualifies compounds if they fall outside specific criteria: a molecule weight outside the range of 200 to 600, a LogP greater than 6, more than six hydrogen donors, over twelve hydrogen acceptors, more than fifteen rotatable bonds, more than seven aromatic rings, fewer than two heteroatoms, or less than five carbon atoms. By applying these criteria, I ensure that only molecules conforming to essential drug-likeness standards progress to further evaluation. This preliminary filtering step is crucial as it assesses molecular properties against established benchmarks, effectively eliminating compounds that are less likely to demonstrate the desired pharmacological profiles. Following this initial filtering, I introduced four distinct scoring functions into the SAGE model. Each function contributes a maximum of one point, cumulatively reflecting a comprehensive assessment of the molecule's characteristics.

Score 1 exclusively focuses on target specificity, leveraging the predictive power of the QSAR model for each protein target. Ranging from 0 to 1, this score quantifies the likelihood of a molecule being an effective inhibitor for a given protein target. For the dual inhibition task (AChE/MAOB), Score 1 is determined by averaging the prediction scores from both targets. This approach ensures a balanced assessment of molecules aimed at multiple targets. The values obtained from my QSAR classification models provide a quantitative measure of the likelihood that a molecule will act as an effective inhibitor. These probabilities reflect the probability that a molecule's binding affinity to its protein target is greater than 1 µM. This probability is a crucial indicator of a molecule’s potential as an inhibitor, ensuring that the SAGE model prioritizes compounds with a higher likelihood of being potent and specific to the intended targets.

Score 2 builds upon the foundation established by Score 1, integrating the concept of synthetic accessibility into the assessment. This score, with a range from 0 to 2, is calculated by summing the target specificity score (Score 1) and the synthetic accessibility measure. For determining synthetic accessibility, I employed the retrosynthetic accessibility score (RAscore), a metric that enables the rapid estimation of a molecule’s synthetic feasibility [22]. RAscore ranges from 0 to 1 and assesses the likelihood of successfully identifying retrosynthetic routes for a molecule using the AiZynthFinder tool [23]. A score closer to 1 indicates a higher probability of finding feasible retrosynthetic pathways, reflecting the molecule’s ease of synthesis.

Score 3 further advances the evaluation process by adding the concept of solubility. This score, with a range from 0 to 3, is derived by summing up Score 2 with the apparent solubility of the molecule. To accurately and efficiently predict aqueous solubility, I utilized the molecule attentions transformer (SolTranNet) [24]. SolTranNet is adept at predicting the solubility of small organic molecules in water, thereby facilitating the identification of compounds with favorable solubility properties. In this context, the solubility measure is quantified in a binary manner: a score of 1 is assigned if the generated molecule is soluble, and a score of 0 if it is not. The determination of a molecule's solubility is based on its LogS value. Typically, a molecule is considered soluble if its LogS falls within the range of − 4 to 0.5 log mol/L [24]. However, due to the relatively high false discovery rate of SolTranNet at the − 4 threshold (28.6%), I have adopted a more flexible threshold of − 6, which significantly reduces the false discovery rate to 1.3% [24].

Score 4 represents the advanced stage of my comprehensive molecule assessment, where I integrated pharmacokinetic aspects through ADME/T profiling. This score, with a range of 0 to 4, is calculated by combining the value of Score 3 with the ADME/T profile score. However, the inclusion of the ADME/T profile score with the ADME/T profile score within Score 3 is subject to two specific conditions. Firstly, all scores of target specificity, synthetic accessibility, and solubility must exceed a threshold of 0.75. This criterion ensures that ADME/T profiling is typically conducted on molecules that have already shown substantial potential efficacy. Secondly, the ADME/T profile score is only added if the QSAR score for the human Ether-à-go-go-Related Gene (hERG) inhibition remains below a critical threshold of 0.5. Given the crucial role of hERG in maintaining heart rhythm, its inhibition can lead to significant adverse effects, where molecules with a binding affinity (IC50) of 40 µM or stronger were classified as positive while those weaker than 40 µM were classified as negative in the dataset [25], thus making it a vital consideration in evaluating drug safety, especially concerning cardiac health.

The ADME/T profile score is a key component of my comprehensive molecule assessment and includes 11 QSPR models for single-target tasks and 12 for the dual inhibitor task. The AChE/MAOB dual task includes an additional indicator for the blood–brain barrier (BBB) permeability. In my ADME/T profile evaluation, I employed QSPR models to predict a range of pharmacokinetic and toxicity properties. Each indicator in the ADME/T profile, ranging from 0 to 1, is integral in determining a molecule’s overall suitability as a therapeutic agent. To calculate the ADME/T profile score, I averaged the scores from all relevant QSPR models. For single-target tasks, I used 11 QSPR models, each contributing approximately 2.27% to Score 4. For the dual-target task, I averaged 12 QSPR models, with each contributing around 2.08% to Score 4. This approach ensures the score remains within a maximum of 1, providing a consistent and balanced evaluation across different tasks.

For absorption, I assessed Caco-2 membrane permeability (Caco2), human intestinal absorption (HIA), and P-glycoprotein inhibition (Pgp). The Caco-2 cell line is used to estimate drug permeation through intestinal tissue, where the maximum value is − 3.51, the minimum value is − 7.76, and the average value is − 5.24 in the dataset [26]. A compound is generally considered to have proper permeability if its predicted Caco2 permeability exceeds − 5.15 log cm/s [27], so I have set − 5.15 log cm/s as the threshold for determining acceptable permeability in my assessment. HIA is crucial for drug delivery through the gastrointestinal system to the intended target, where molecules with intestinal fraction absorption of 30% or higher were classified as positive while those below 30% were classified as negative in the dataset [28]. Pgp is an ABC transporter responsible for transporting substances in and out of cells, and its inhibition can impact a drug’s bioavailability and safety, where the molecules with a binding affinity (IC50) stronger than 15 µM were classified as positive while those with binding affinity weaker than 100 µM were classified as negative in the dataset [29].

In evaluating distribution, I considered the human plasma protein binding rate (PPBR) and BBB permeability. PPBR measures the proportion of a drug bound to plasma proteins in the bloodstream, and the rate significantly affects the drug’s delivery to its target, where the maximum value is 99.95, the minimum value is 10.09, and the average value is 86.73 in the dataset [30]. A compound is generally classified as having high binding if its PPBR exceeds 80%. I have established 80% as the threshold for acceptable plasma protein binding, ensuring that compounds meet this criterion for effective drug delivery. BBB serves as a protective layer separating the circulating blood from the extracellular fluid in the brain, where molecules with BBB penetration partition of − 1 or higher were classified as positive while those lower than − 1 were classified as negative in the dataset [31]. Its penetration is a critical factor in drug delivery, as a molecule must pass through the BBB to reach its intended target within the brain.

For metabolism, my focus is on the inhibition of cytochrome P450 genes (CYP2C9, CYP2D6, and CYP3A4). These genes are necessary for the metabolism or breakdown of many molecules within cells, and if a drug can block these enzymes, it may result in poor metabolism, where molecules showing no response at concentrations up to 57 µM were classified as negative while those exhibiting a response were classified as positive in the dataset [32]. In toxicity assessment, I considered lethal dose 50 (LD50), hERG inhibition, mutagenicity (AMES), and drug-induced liver injury (DILI). LD50 measures rat acute toxicity and determines the lowest dose of a substance that can cause lethal side effects, where the maximum value is 10.207, the minimum value is − 0.343, and the average value is 2.544 in the dataset [33]. A compound is classified as acutely toxic if its LD50, determined via rodent oral administration, is below 300 mg/kg. Assuming a molecular weight of 300, this LD50 corresponds to 5 log(1/mol/kg), a value I have adopted as the threshold criterion. AMES refers to a drug’s capacity to harm DNA and lead to cell death or other negative consequences, where molecules, which increased the number of revertant colonies per plate in a dose-related manner in the Ames test, were classified as positive while those that do not were classified as negative in the dataset [34]. DILI is a serious liver disease that can be caused by certain drugs, where molecules with a high risk of DILI or the potential to cause any adverse liver effects in their annotations were classified as positive while those with no risk of DILI or not associated with any adverse liver effects in the dataset [35].

### Data sets for target specificity and ADME/T prediction

The ligand structures used for target specificity were obtained from the DUD-E benchmarking sets [36] and ChEMBL32 [18], as summarized in Table 1. To ensure a comprehensive evaluation of my SAGE program’s applicability across diverse biological functions and structures, I strategically selected six protein targets: Acetylcholinesterase (AChE), Cyclooxygenase-2 (COX-2), Protein kinase C beta (PKCB), Fibroblast growth factor receptor 1 (FGFR1), Protein-tyrosine phosphatase 1B (PTP1B), and Monoamine oxidase B (MAOB). These targets were chosen not only for their evenly distributed active and inactive experimental data, which helps to avoid bias from unbalanced datasets, but also because they represent a diverse range of functional classes in pharmacological contexts. This variety in selection underscores the versatility and potential broad applicability of the SAGE program, demonstrating its effectiveness across different types of protein targets with unique structural and functional attributes.

To create QSAR models for predicting ADME/T properties, I gathered ligand structures from Therapeutics Data Commons [37], which are summarized in Table 1. I selected 12 ADME/T properties, including Caco2 [26], HIA [28], Pgp [29], PPBR [30], BBB [31], CYP2D6 [32], CYP3A4 [32], CYP2C9 [32], LD50 [33], hERG [25], AMES [34], and DILI [35], with three (Caco2, PPBR, and LD50) being predicted as regression tasks and the remaining nine as classification tasks.

### Molecular fingerprints and machine learning

To quantify the structural similarity of chemical compounds, I used two-dimensional (2D) chemical fingerprints as binary features. Firstly, I used predefined 2D chemical fingerprint dictionaries, which were designed for the analysis of large chemical libraries. The molecular access system (MACCS) is one of the most frequently used fingerprint schemes for quantifying similarity with 166-bit MACCS keys [38], while the PubChem system utilizes substructure fingerprints (PCFP) to represent chemical structures and enable similarity searching and neighboring with 881 structural keys [39]. Secondly, atom-connectivity fingerprints in the molecules were considered. The extended-connectivity fingerprints (ECFP) were designed for structure–activity modeling and representing circular atom neighborhoods rather than substructure and similarity searching [40]. Function-class fingerprints (FCFP) are a type of ECFP-based fingerprints but have different indexing of the roles of specific atoms in the environment. Because the FCFP does not distinguish between different atoms or groups with the same or similar function, it can be used for pharmacophore-like fingerprints. I generated the ECFP with a diameter of 6 (ECFP6) and FCFP with a diameter of 4 (FCFP4) as 1024 bits using RDKit [41] and Morgan algorithms [42]. By concatenating MACCS keys and three fingerprints (PCFP, ECFP6, and FCFP4), the more complex fingerprints (MACCS + PCFP, MACCS + ECFP6, and MACC + FCFP4) were generated and used as features for QSAR models.

To develop QSAR models for target specificity, I used the Scikit-learn package [43] in Python to perform a stratified split of the data, with 80% of the compounds used for training and 20% for test sets, while maintaining a fixed random seed. For QSAR models related to ADME/T, I employed a scaffold-based split in Therapeutics Data Commons [37]. During the training phase, I used tenfold cross-validation with GridSearchCV in the Scikit-learn package [43]. To evaluate classification tasks on the test sets, I used various metrics such as AUC, Precision, Recall, and F1-score. AUC measures the area under the receiver operating characteristic curve, which shows the model's ability to distinguish between classes. Precision, or positive predictive value, indicates the proportion of true positives among the predicted positives. Recall is sensitivity or true positive rate and represents how many of the actual positives the model correctly identified as positive. F1-score is a harmonic average of precision and recall and is a better measure to use if a balance between precision and recall is needed. Additionally, a threshold value of 0.5 was used in the classification model to decide the class labels.

I utilized four ensemble-based machine learning algorithms: gradient boosting machine (GBM), light gradient boosting machine (LGBM), random forest (RF), and extreme gradient boosting (XGB). These algorithms use decision trees to prevent overfitting and reduce variance, with each decision tree analyzing numerical features to generate continuous outputs. The decision trees are constructed sequentially and adjusted to the differences between actual and predicted values generated by previous trees.

The hyperparameter tuning parameters are summarized in Additional file 1: Table S1. I conducted a grid search to find the best model in four hyperparameters in GBM, two in LGBM, and two in RF and XGB. In GBM, I used four hyperparameters for tuning the models: the number of gradient-boosted trees (n_estimators), the maximum tree depth for base learners (max_depth), the number of features to consider when selecting the best split (max_features), and the boosting learning rate (learning_rate). In LGBM, I used two hyperparameters: the number of gradient-boosted trees (n_estimators) and the boosting learning rate (learning_rate). In RF, I used two hyperparameters: the number of gradient-boosted trees (n_estimators) and the number of features to consider when selecting the best split (max_features). In XGB, I used three hyperparameters: the number of gradient-boosted trees (n_estimators), the maximum tree depth for base learners (max_depth), and the boosting learning rate (learning_rate).

I conducted a tenfold cross-validation and grid search to find the optimal values of each set of descriptors. The optimal hyperparameters were determined based on the model performance in the cross-validation sets, and I selected the 12 optimal models for each task. The best model was selected from the 12 optimal models based on the geometric mean of two performances in the cross-validation and test sets.

### Molecular simulations

X-ray crystal structures of human AChE (PDB ID: 6NTO [44]) and human MAOB (PDB ID: 1S3B [45]) were obtained from the Protein Data Bank (PDB) [46]. Missing side chains were predicted using Prime [47], and hydrogen atoms were added to these protein structures at a pH of 7.0. The coordinates of these atoms were subsequently optimized with PROPKA3 [48]. The restrained energy minimization was performed with OPLS3 within 0.3 Å root-mean-square deviation (RMSD) [49].

Molecular docking was performed using Glide-SP in Prime [50], selecting the docking poses with the highest docking scores for dual targets (AChE and MAOB). The protein–ligand complexes of AChE with Ladostigil and the top-ranked molecule were incorporated into an orthorhombic box containing 15,329 and 15,303 water molecules (TIP3P model), respectively, as generated by a 10 Å buffer distance. The protein–ligand complexes of MAOB with Ladostigil and the top-ranked molecule were similarly inserted into an orthorhombic box with 15,930 and 15,305 water molecules, respectively. To achieve a neutral system and simulate a physiological concentration of 0.15 M, the AChE system with Ladostigil incorporated 52 Na^+^ and 43 Cl^−^ ions, while the AChE system with the top-ranked molecule included 54 Na^+^ and 43 Cl^−^. Similarly, the MOAB system with Ladostigil involved 47 Na^+^ and 44 Cl^−^ ions, whereas the MAOB system with the top-ranked molecule incorporated 48 Na^+^ and 43 Cl^−^ ions.

Molecular dynamics simulations were conducted employing Desmond [51] using an OPLS3 force field and an NVT ensemble, ensuring a constant number of particles, volume, and temperature. The Particle-mesh Ewald method was applied to compute long-range and short-range interactions with a cutoff of van der Waals and electrostatic interactions of 9 Å [52]., Nose–Hoover thermostats were employed to maintain a constant temperature of 300 K [53]. The RESPA integrator was utilized to combine the equations of motion with a time step of 2.0 fs for bonded and non-bonded interactions [54]. A 50 ns simulation was conducted using the default Desmond protocol, and the conformations and energies were stored at 50 and 1.2 ps intervals, respectively. For analysis, only conformations extracted from the 10 to 40 ns timeframes were considered, thereby excluding potentially unstable conformations from the initial and terminal phases of the simulations. I measured the RMSD values with heavy atoms of the protein and ligand structures based on a reference frame.

## Results

### Scoring-assisted generative exploration (SAGE)

**Fig. 1:**
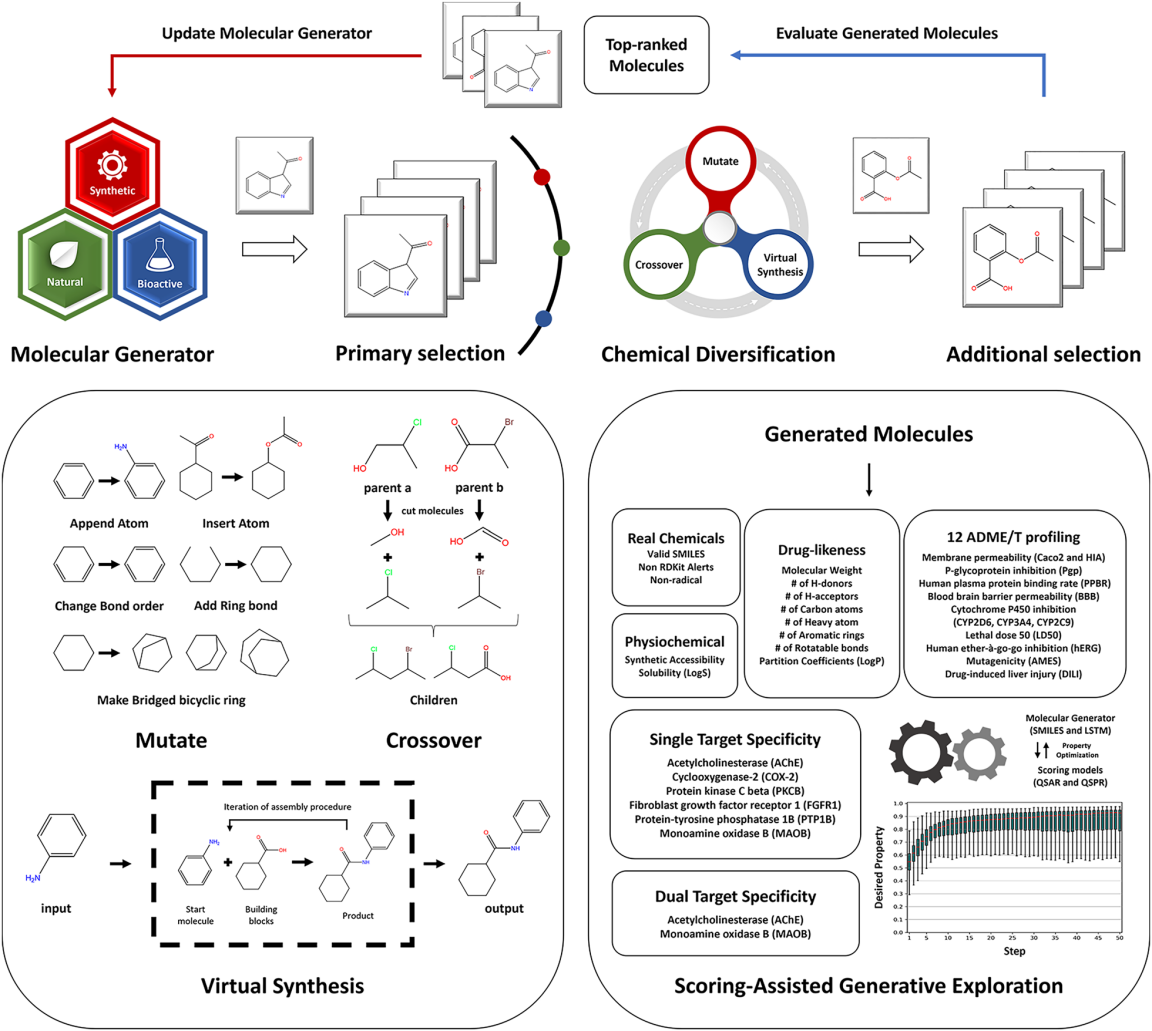
Scoring-assisted generative exploration (SAGE)

Scoring-assisted generative exploration (SAGE) combines deep neural networks (DNN), genetic improvement operators, and multiple scoring models to generate highly rewarding molecules through iterative fine-tuning (Fig. 1). To enhance the generation of structurally diverse compounds, I have expanded the capabilities of SAGE by incorporating features that enable virtual synthesis simulation and the creation of bridged bicyclic rings. The effectiveness of the SAGE algorithms was validated with three goal-directed benchmarks and the datasets used in this study are summarized in Table 1. I introduced four distinct models named M100, M75/V20/B05, M50/V45/B05, and M25/V70/B05 for the newly added operators, each based on their probability distribution of the newly added operators. Notably, the M100 operates identically to the original GEGL.

Firstly, I applied the SAGE algorithms to general goal-directed benchmarks in GuacaMol [20], with the summarized results in Table 2. For the GuacaMol benchmark, I initialized the LSTM models using the weights provided by Brown et al. [20], which were pre-trained with the ChEMBL24 database [18]. In the GuacaMol benchmark, the performance scores for M100, M75/V20/B05, M50/V45/B05, and M25/V70/B05 were 16.401, 16.507, 16.443, and 16.458, respectively, demonstrating an improvement over the scores achieved by two baselines without the ranking-based fine-tuning mechanism (a reward system), including SMILES LSTM (11.258) and SMILES GA (9.440). Both M100 and my SAGE models incorporate elements from SMILES LSTM and SMILES GA, with my SAGE models showing equal or superior performance compared to the highest scores obtained by M100, SMILES LSTM, and SMILES GA in the GuacaMol benchmark tasks.

**Table 2:** Results of the SAGE models for the goal-directed benchmarks in GuacaMol

Task	Target Compound	SMILES LSTM^a^	SMILES GA^a^	M100 (GEGL)	M75/V20/B05	M50/V45/B05	M25/V70/B05
Rediscovery	Celecoxib	0.609	0.506	**1.000**	**1.000**	**1.000**	**1.000**
	Troglitazone	0.465	0.333	**1.000**	**1.000**	**1.000**	**1.000**
	Thiothixene	0.544	0.433	**1.000**	**1.000**	**1.000**	**1.000**
Similarity	Aripiprazole	0.740	0.609	**1.000**	**1.000**	**1.000**	**1.000**
	Albuterol	0.835	0.644	**1.000**	**1.000**	**1.000**	**1.000**
	Mestranol	0.822	0.466	**1.000**	**1.000**	**1.000**	**1.000**
Isomer	C_11_H_24_	0.716	0.872	**1.000**	**1.000**	**1.000**	**1.000**
	C_9_H_10_N_2_O_2_PF_2_Cl	0.738	0.769	**1.000**	**1.000**	**1.000**	0.984
Multiple Property Optimization	Fexofenadine	0.794	0.752	**1.000**	**1.000**	**1.000**	**1.000**
	Ranolazine	0.799	0.758	0.948	0.948	**0.955**	0.951
	Perindopril	0.556	0.519	0.848	0.882	0.845	**0.883**
	Amlodipine	0.690	0.622	0.906	0.913	**0.924**	**0.924**
	Sitagliptin	0.494	0.470	0.912	0.926	**0.929**	0.925
	Zaleplon	0.540	0.510	0.788	**0.838**	0.795	0.792
Valsartan SMARTS		0.334	0.027	0.999	**1.000**	0.997	0.999
Decorator Hopping		0.912	0.624	**1.000**	**1.000**	**1.000**	**1.000**
Scaffold Hopping		0.670	0.525	**1.000**	**1.000**	**1.000**	**1.000**
Total		11.258	9.440	16.401	**16.507**	16.443	16.458

M is a mutate operator, V is a virtual synthesis operator, and B is a bridged bicyclic ring operator

^a^Without the ranking-based fine-tuning mechanism

Bold value indicates the best results

In contrast to the M100, my SAGE models can generate more complex molecules due to the addition of virtual synthesis and bridged bicyclic ring operators. Virtual synthesis operators allow for the exploration of a broader chemical space by simulating potential synthetic pathways, which can lead to novel molecular scaffolds. The inclusion of bridged bicyclic ring operators specifically aids in constructing more intricate ring systems, which are often found in bioactive compounds. These enhancements in molecular complexity are advantageous for MPO tasks that require balancing multiple physicochemical properties simultaneously. This is evident in MPO tasks involving compounds like Perindopril, Amlodipine, Sitagliptin, and Zaleplon in Table 2, which are octahydroindole, dihydropyridine, triazolopiperazine, and pyrazolopyrimidine derivatives, respectively, demanding complex molecular structures. The Amlodipine task focused on maintaining three carbon rings, and the Sitagliptin and Zaleplon tasks involved generating isomers. In these intricate tasks, where maintaining a specific number of rings or generating isomers while optimizing other properties is crucial, my SAGE achieved a higher score than that of M100. This enhanced performance is attributed to the increased complexity that the virtual synthesis and bridged bicyclic ring operators add to the molecule generation process.

Secondly, in my exploration of SAGE’s abilities, I paid particular attention to its performance on the rediscovery and similarity tasks, where the SAGE should generate compounds resembling a specified target molecule removed from the training set. I further validated the ability of SAGE to discover eleven compounds with structurally complex bridged bicyclic rings, which is summarized in Table 3. For the bridged bicyclic ring benchmark, I initialized the LSTM models using the weights provided by Ahn et al. [7], which were pre-trained with the ZINC database [55]. As a result, the M75/V20/B05 showed the best score of 21.141 by more effectively generating bridged bicyclic ring structures, while the M100 achieved a score of 20.790. Moreover, in the benchmark tasks in Tables 2 and 3, the M75/V20/B05 did not record a lower score than the M100 in a single instance. It indicates that SAGE is not only capable of replicating the functionalities of the M100 but is also adept at de novo design for more structurally complex molecules than the M100. Therefore, I employed the M75/V20/B05 probability for SAGE.

**Table 3:** Results of rediscovery and similarity tasks for bridged bicyclic ring structures

Task	Target Compound	M100 (GEGL)	M75/V20/B05	M50/V45/B05	M25/V70/B05
Rediscovery	Ingenol mebutate	0.732	**0.798**	0.732	0.732
	Morphine	0.676	**0.707**	0.632	0.676
	Amantadine	**1.000**	**1.000**	**1.000**	**1.000**
	Rimantadine	**1.000**	**1.000**	**1.000**	**1.000**
	Vildagliptin	**1.000**	**1.000**	**1.000**	**1.000**
	Memantine	**1.000**	**1.000**	**1.000**	**1.000**
	Tromantadine	**1.000**	**1.000**	**1.000**	**1.000**
	Adapalene	**1.000**	**1.000**	**1.000**	**1.000**
	Saxagliptin	**1.000**	**1.000**	**1.000**	**1.000**
	Azaprophen	0.790	**1.000**	**1.000**	**1.000**
	Psiguadial A	0.767	0.787	0.787	**0.798**
Similarity	Ingenol mebutate	0.976	**1.000**	0.976	0.893
	Morphine	0.935	0.935	0.901	**1.000**
	Amantadine	**0.918**	**0.918**	0.913	0.912
	Rimantadine	**1.000**	**1.000**	**1.000**	**1.000**
	Vildagliptin	**1.000**	**1.000**	**1.000**	**1.000**
	Memantine	**0.996**	**0.996**	**0.996**	**0.996**
	Tromantadine	**1.000**	**1.000**	**1.000**	**1.000**
	Adapalene	**1.000**	**1.000**	**1.000**	**1.000**
	Saxagliptin	**1.000**	**1.000**	**1.000**	**1.000**
	Azaprophen	**1.000**	**1.000**	**1.000**	**1.000**
	Psiguadial A	**1.000**	**1.000**	**1.000**	**1.000**
Total		20.790	**21.141**	20.937	21.007

M is a mutate operator, V is a virtual synthesis operator, and B is a bridged bicyclic ring operator

Bold value indicates the best results

### Pre-training the SAGE models with four databases

To validate the suitability of SAGE for de novo design, I applied the SAGE algorithm to identify new potential inhibitors for a target protein. I pre-trained SAGE on four datasets (ChEMBL24, Synthetics, ZINC, and ZINC-NP), the details of which are summarized in Table 1. Then I generated 1000, 3000, and 5000 molecules for each dataset with the pre-trained SAGE, which are summarized in Additional file 1: Table S2. I employed commonly used four metrics in de novo design for comparison (validity, uniqueness, novelty, and internal diversity). Firstly, all pre-trained models showed high validity and high uniqueness scores, which indicates that all models can generate minimal duplicate molecules. Secondly, the novelty metric increased in the order of ZINC-NP, ChEMBL24, Synthetics, and ZINC. A lower value in this metric suggests that the pre-trained model is generating molecules similar to those found in the training set. The natural product (ZINC-NP) and bioactive compounds (ChEMBL24) datasets led to the generation of molecules that reflect the characteristics inherent to the training sets. It indicates that the DNN is learning the features of natural products or bioactive compounds through the pre-training process, which is an expected outcome. Given my objective to generate new potential inhibitors, not present in the training set, for a target protein, I selected synthetic compounds. Thirdly, I compared internal diversity scores between Synthetics and ZINC databases, where the model pre-trained with Synthetics showed a better score than that with ZINC. Based on these outcomes, I employed the SAGE, which is pre-trained on commercially available synthetic compounds (Synthetics).

### QSAR models for target specificity and single property optimization with SAGE

**Fig. 2:**
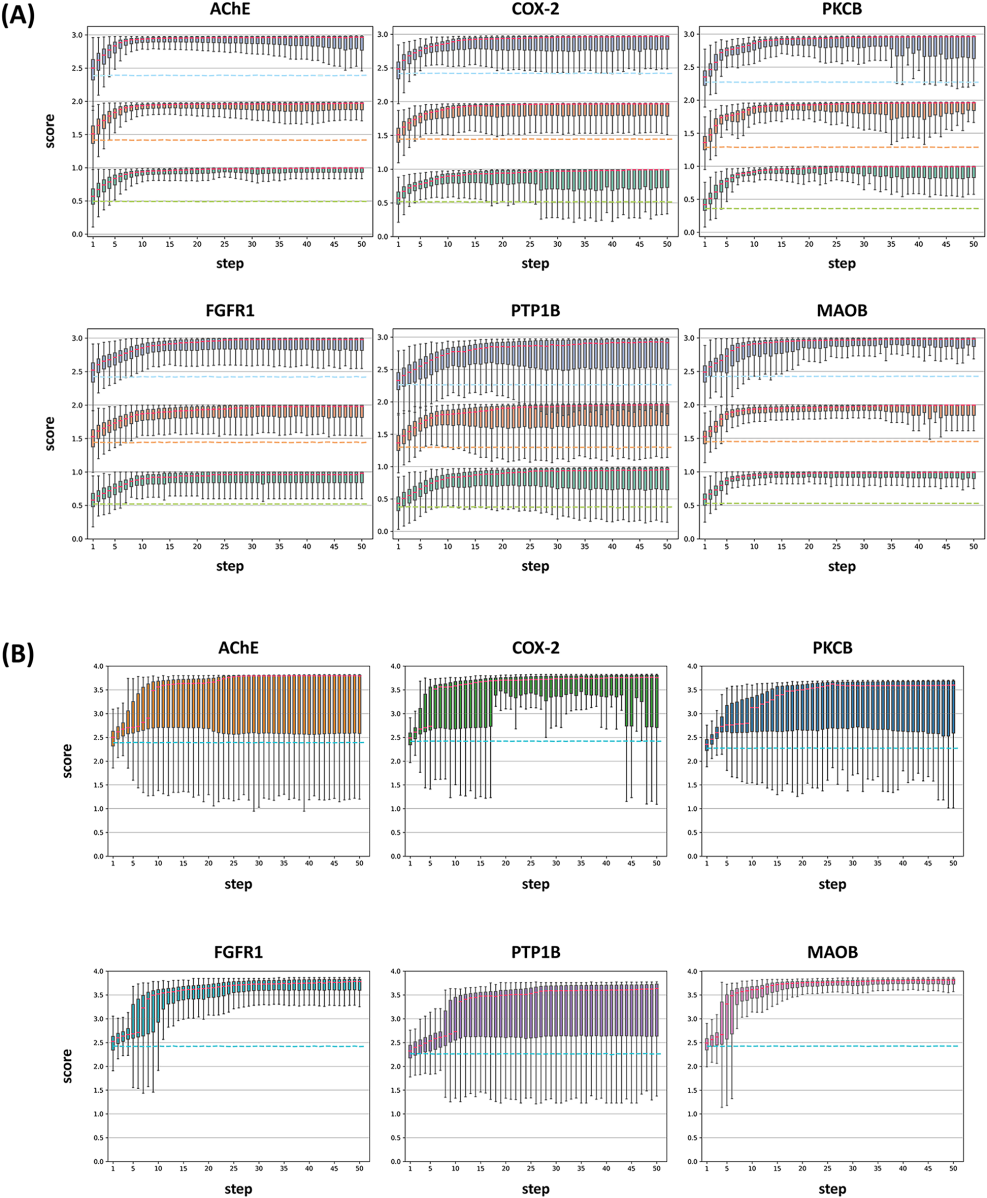
SAGE-based Single Target Specificity Optimization for Six Targets. **A** A series of boxplots are represented, depicting the steps in the SAGE process across six target proteins, as evaluated with Scores 1, 2, and 3. These scores are represented in green, orange, and blue, respectively. The medians of each boxplot are highlighted in red, and the baselines are depicted with dashed lines. **B** For Score 4, a series of boxplots is shown, illustrating the steps in the SAGE process across the same six target proteins. The medians of these boxplots are indicated in red, while the baselines are shown with dashed lines in cyan

After pre-training, I made single property optimization tasks for target specificity. To make QSAR models for target specificity, I selected six protein targets (AChE, COX-2, PKCB, FGFR1, PTP1B, and MAOB), which have balanced sets of experimentally active and inactive ligands and represent a range of different functional classes and possess unique structural and functional characteristics among the protein targets in the DUD-E benchmarks [36]. I fine-tuned the hyperparameters through tenfold cross-validation and selected the best model based on the geometric mean of the AUC scores in cross-validation and test sets (Additional file 1: Tables S3, S4). The performance metrics of the best-found models are summarized in Table 4. These models were then integrated into SAGE for inhibitor design tasks against the six protein targets. To restrict the chemical space into drug-like molecules, I employed Muegge’s drug-likeness for chemical filters. I gradually added extra points for synthetic accessibility (Score 2) and solubility (Score 3), starting with a single target specificity (Score 1) to evaluate the ability of SAGE to design inhibitors (Fig. 2A). As my baseline, I selected each median score of the generated molecules at every step from the SAGE model without the fine-tuning strategy.

**Table 4:** Performance metrics of the best-found QSAR models

Name	Task	Metric	Model	Train Set	Validation set	Test set
AChE	Target Specificity	AUC	MACCS + PCFP/RF	1.000 ± 0.000	0.968 ± 0.022	0.947
COX-2		AUC	MACCS + FCFP4/RF	1.000 ± 0.000	0.789 ± 0.104	0.818
PKCB		AUC	MACCS + FCFP4/RF	1.000 ± 0.000	0.941 ± 0.070	0.894
FGFR1		AUC	MACCS + ECFP6/RF	1.000 ± 0.000	0.954 ± 0.064	0.930
PTP1B		AUC	MACCS + PCFP/RF	1.000 ± 0.000	0.944 ± 0.069	0.947
MAOB		AUC	MACCS + ECFP6/RF	1.000 ± 0.000	0.901 ± 0.085	0.842
Caco2	Absorption	MAE	MACCS + FCFP4/RF	0.125 ± 0.002	0.391 ± 0.047	0.348
HIA		AUC	MACCS + PCFP/XGB	0.988 ± 0.002	0.935 ± 0.055	0.889
Pgp		AUC	MACCS + FCFP4/RF	1.000 ± 0.000	0.900 ± 0.049	0.874
BBB	Distribution	AUC	MACCS + FCFP4/XGB	0.967 ± 0.002	0.878 ± 0.041	0.807
PPBR		MAE	MACCS + FCFP4/RF	3.736 ± 0.088	10.926 ± 1.414	9.126
CYP2D6	Metabolism	AUC	MACCS + FCFP4/LGBM	0.858 ± 0.002	0.820 ± 0.016	0.795
CYP3A4		AUC	MACCS + FCFP4/XGB	0.925 ± 0.002	0.854 ± 0.030	0.811
CYP2C9		AUC	MACCS + PCFP/LGBM	0.865 ± 0.002	0.834 ± 0.014	0.795
LD50	Toxicity	MAE	MACCS + PCFP/RF	0.154 ± 0.001	0.450 ± 0.047	0.575
hERG		AUC	MACCS + FCFP4/RF	1.000 ± 0.000	0.820 ± 0.069	0.717
AMES		AUC	MACCS + FCFP4/RF	1.000 ± 0.000	0.822 ± 0.064	0.776
DILI		AUC	MACCS + FCFP4/XGB	0.965 ± 0.004	0.854 ± 0.082	0.861

AChE breaks down acetylcholine, but its inhibitor prevents this breakdown and increases neurotransmitter levels in the brain [56]. The MACCS + PCFP/RF model for AChE showed the best performance (${\text{AUC}}=0.947$), with precision, recall, and F1-score of 0.945 in test sets. To maximize target specificity, I combined the best model for AChE with SAGE and performed iterative fine-tuning with 50 steps. For Score 1, the SAGE achieved a median score of over 0.75 from the 4th step and over 0.90 from the 7th step. For Score 2, the SAGE achieved a median score of over 1.75 from the 4th step and over 1.90 from the 7th step. For Score 3, the SAGE achieved a median score of over 2.75 from the 4th step, with a median score of over 2.90 from the 7th step.

COX-2 is an enzyme that converts arachidonic acid to prostaglandin endoperoxide H2, and selective inhibitors of COX-2 can lower the risk of peptic ulceration [57]. The MACCS + FCFP4/RF model for COX-2 showed the best performance (${\text{AUC}}=0.818$) in grid search, with precision, recall, and F1-score of 0.816 in test sets. I used SAGE and performed iterative fine-tuning with 50 steps to optimize Score 1, 2, and 3 for COX-2. For Score 1, the SAGE achieved a median score of over 0.75 from the 4th step, while it found molecules with median scores over 0.90 from the 11th step. For Score 2, the SAGE achieved a median score over 1.75 from the 5th step, with a median score over 1.90 from the 11th step. When optimizing Score 3, the SAGE achieved a median score higher than 2.75 from the 5th step, while it showed a median score over 2.90 from the 12th step.

PKCB is a crucial protein in the maintenance of nerve functions, and inhibiting its activity has the potential as a tumor treatment [58]. The MACCS + FCFP4/RF model performed best for PKCB, with an AUC of 0.894 and an F1-score of 0.885 in test sets. By combining this model with SAGE, I conducted iterative fine-tuning with 50 steps to optimize Score 1. The SAGE achieved a median score of over 0.75 in the median from the 5th step and a median score of over 0.90 from the 9th step. When I conducted iterative fine-tuning for Score 2 and Score 3 with 50 steps, the SAGE for Score 2 achieved a median score of over 1.75 from the 5th step and a median score of over 1.90 from the 12th step. For Score 3, the SAGE achieved a median score of over 2.75 from the 5th step and a median score higher than 2.90 from the 12th step.

The deregulation of FGFR1 signaling is associated with various human cancers, and targeted inhibitors of this pathway have proven successful in tumor therapy [59]. The MACCS + ECFP4/RF model showed the best performance for FGFR1 with an AUC of 0.930, precision, recall, and F1-score of 0.929 in test sets. Using SAGE with 50 steps, I optimized Score 1, 2, and 3 for FGFR1, achieving median scores over 0.75 from the 5th step and over 0.90 from the 10th step for Score 1. For Score 2, the SAGE achieved a median score over 1.75 from the 6th step and 1.90 from the 16th step, while for Score 3, it found molecules over 2.75 from the 7th step and over 2.90 from the 14th step in the median.

PTP1B overexpression can cause a decrease in insulin receptor phosphorylation, and mutations in the PTP1B gene can lead to diabetes, making its inhibitor a potential diabetes treatment [60]. The MACCS + PCFP/RF model demonstrated the best performance for PTP1B, achieving an AUC of 0.947, a precision of 1.000, a recall of 0.885, and an F1-score of 0.939 in test sets. Using the SAGE to optimize Score 1, 2, and 3 for PTP1B, I found that the SAGE identified molecules with a median score over 0.75 from the 8th step and those over 0.90 from the 21st step. Similarly, when the desired property was changed to Score 2, the SAGE generated molecules with median scores over 1.75 from the 11th step and those over 1.90 from the 20th step. For Score 3, the SAGE achieved a median score of over 2.75 from the 11th step and over 2.90 from the 14th step.

MAOB is an enzyme that breaks down brain chemicals, such as dopamine, and its inhibitors of this enzyme can increase dopamine availability in the brain [61]. The MACCS + ECFP6/RF model showed the best performance for MAOB, with an AUC of 0.842, a precision of 0.870, a recall of 0.800, and an F1-score of 0.833 in test sets. Using iterative fine-tuning with SAGE and the best model for MAOB, I optimized Score 1, 2, and 3. The SAGE for Score 1 achieved a median score of over 0.75 from the 4th step and over 0.90 from the 6th step. For Score 2, the SAGE generated molecules with median scores over 1.75 from the 4th step, and over 1.90 from the 7th step. Finally, the SAGE for Score 3 identified molecules with median scores over 2.75 from the 6th step and over 2.90 from the 10th step.

The SAGE algorithm was used to optimize target specificity for six protein targets, achieving median scores over 0.75 within 5 steps and higher than 0.9 within 10 steps for Score 1. Synthetic accessibility was then added to the desired property and the SAGE for Score 2 generated molecules with median scores over 1.75 and 1.90 within 5 and 12 steps, respectively, for the six targets. Similarly, when considering solubility in addition to target specificity and synthetic accessibility, the SAGE for Score 3 found molecules with median scores of over 2.75 within 6 steps and higher than 2.90 within 14 steps. For generating top-ranked molecules, the SAGE found molecules over 0.90 for Score 1 in the 1st step, over 1.90 for Score 2 in the 2nd step, and over 2.90 for Score in the 3rd step, on average across the six targets.

### QSAR models for ADME/T and multiple property optimization with SAGE

Drug candidates’ success depends on their ADME/T profile in addition to target specificity, synthetic accessibility, and solubility. With the accumulation of experimental data and the development of in silico prediction models, predicting ADME/T properties has become easier. Therefore, I selected 12 ADME/T properties (Caco2, HIA, Pgp, BBB, PPBR, CYP2D6, CYP3A4, CYP2C9, LD50, hERG, AMES, and DILI) [37] and developed QSAR models for each. I fine-tuned the hyperparameters and selected the best-found models similar to the QSAR models for target specificity. The performance metrics of the best-found models for each ADME/T property are summarized in Table 4, while the performance metrics and optimal hyperparameters of all models are summarized in Additional file 1: Tables S5–S8.

The MACCS + FCFP4/RF for Caco2 (${\text{MAE}}=0.348$ and $R=0.776$), MACCS + PCFP/XGB for HIA (${\text{AUC}}=0.889$), and MACCS + FCFP4/RF for Pgp (${\text{AUC}}=0.874$) were selected for absorption prediction. The MACCS + FCFP4/XGB for BBB (${\text{AUC}}=0.807$) and MACCS + FCFP4/RF for PPBR (${\text{MAE}}=9.126$ and $R=0.479$) were used to predict distribution. The MACCS + FCFP4/LGBM for CYP2D6 (${\text{AUC}}=0.795$), MACCS + FCFP4/XGB for CYP3A4 (${\text{AUC}}=0.811$), and MACCS + PCFP/LGBM for CYP2C9 (${\text{AUC}}=0.795$) were selected for metabolism. The MACCS + PCFP/RF for LD50 (${\text{MAE}}=0.575$ and $R=0.613$), MACCS + FCFP4/RF for hERG (${\text{AUC}}=0.717$), MACCS + FCFP4/RF for AMES (${\text{AUC}}=0.776$), MACCS + FCFP4/XGB for DILI (${\text{AUC}}=0.861$) were used to predict toxicity.

Similar to the optimization of single target specificity with SAGE, I evaluated the ability of SAGE for MPO tasks of target specificity, synthetic accessibility, solubility, and 11 ADME/T properties, the results of which are illustrated in Fig. 2B.

When the SAGE was employed to maximize Score 4 for AChE, it achieved a score of over 3.5 in the first step of the max and from the 10th step in the median. Moreover, a score of over 3.7 was attained from the second step of the max and the 23rd step in the median. Secondly, the SAGE was also used for COX-2, where it achieved a score of over 3.5 in the first step of the max and from the 6th step in the median. In addition, a score of over 3.7 was obtained from the 4th step of the max and the 19th step in the median. Thirdly, in PKCB, the SAGE obtained a score of over 3.5 from the third step of the max and the 20th step in the median. Additionally, it attained a score of over 3.7 from the 4th step of the max but did not achieve a score of over 3.7 in the median. Fourthly, similarly, for FGFR1, the SAGE achieved a score of over 3.5 in the first step of the max and from the 10th step in the median. It also obtained a score of over 3.7 from the 6th step of the max and the 25th step in the median. Fifthly, in PTPB1, the SAGE attained a score of over 3.5 from the 5th step of the max and the 21st step in the median. Then, it achieved a score of over 3.7 from the 17th step of the max but did not obtain a score of over 3.7 in the median. Lastly, for MAOB, the SAGE achieved a score of over 3.5 in the first step of the max and the 7th step in the median. It also obtained a score of over 3.7 from the third step of the max and the 14th step in the median. As a result, the SAGE achieved a score of over 3.7 in all six targets, with the best scores being 3.82 for AChE, 3.826 for COX-2, 3.706 for PKCB, 3.864 for FGFR1, 3.777 for PTPB1, and 3.868 for MAOB in 50 steps.

### Application of SAGE to dual inhibitor design of AChE and MAOB

Multimodal drugs, having multiple targets, offer advantages over traditional drugs, such as reducing the risk of drug resistance, improving efficacy, and reducing side effects. However, computational design is challenging due to the complexity of their mechanism. To apply the SAGE model to dual inhibitor design, I focused on AChE and MAOB proteins, inspired by the multimodal compound Ladostigil. The compound is known for its dual action in Alzheimer's Disease (AD), with IC50 values of 37.1 and 31.8 uM for AChE and MAOB, respectively [11, 62].

**Fig. 3:**
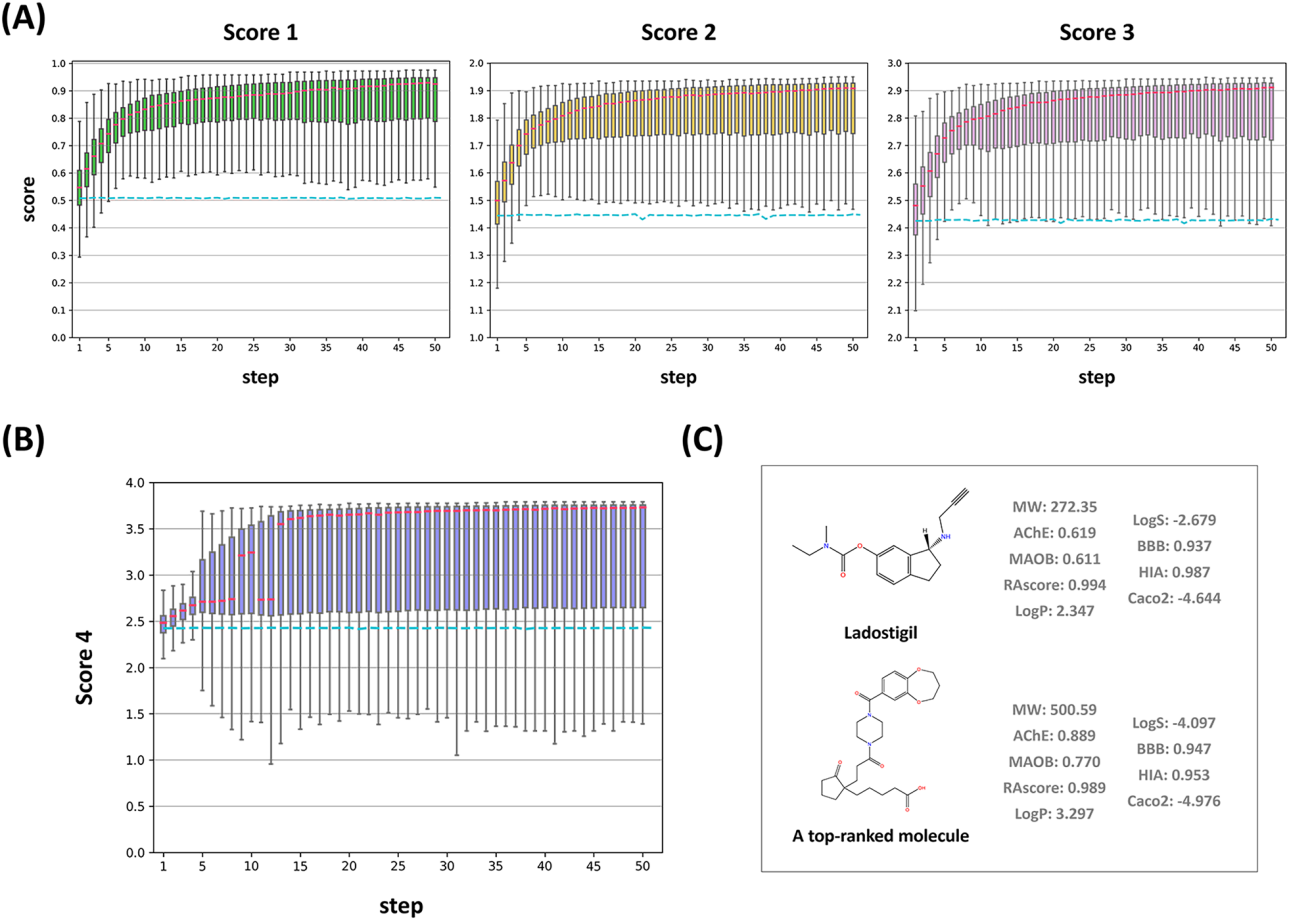
SAGE-based dual target specificity optimization for AChE/MAOB **A** Boxplots for each step of the SAGE process in the AChE/MAOB dual target, categorized Scores 1, 2, and 3, are represented in green, yellow, and pink, respectively. The medians of each boxplot are highlighted in red, while the baselines are depicted with dashed lines in cyan. **B** For Score 4, boxplots depicting each step of the SAGE process in the AChE/MAOB dual target are presented in blue. The medians of each boxplot are highlighted in red, and the baselines are also depicted with dashed lines in cyan. **C** A top-ranked molecule and Ladostigil are illustrated. Their predicted values by SAGE are shown

Similar to my approach in single-target tasks, I engaged in an iterative fine-tuning process with the SAGE model, extending this method over 50 steps for a dual-target task. However, due to the dual-target nature of the task, it was necessary to redefine the target specificity that I previously used for single-target tasks. To determine the target specificity for the dual-target task involving AChE and MAOB, I employed the average of the prediction values from the QSAR models for both AChE and MAOB. My primary objective during this process was to enhance the scores of the molecules generated by the SAGE. To address each score's improvement, I implemented four independent rounds of enhancement within my iterative fine-tuning process. Each round was independently dedicated to elevating one of the scores: Score 1, Score 2, Score 3, and Score 4, respectively. The multiple property optimization of SAGE for dual inhibitor design is shown in Fig. 3A and B. The SAGE achieved a score of over 3.5 from the second step (max) and the 13th step (median), and a score of over 3.7 from the 8th step (max) and the 32nd step (median).

To identify the best molecule for the AChE/MAOB dual targets, I manually filtered all the molecules generated by SAGE. In the 50 steps of maximizing Score 4, SAGE generated 305,535 compounds. Among them, 377 had scores lower than 1, 12,710 had scores between 1 and 2, 169,895 had scores between 2 and 3, and 122,535 had scores between 3 and 4. All 122,535 compounds had already passed the basic cut-off in Muegge’s drug-like chemical space, as defined by Score 4. To pass the 3-point threshold, a compound must have scores greater than 0.75 for average activity (Score 1), synthetic accessibility, and solubility. Secondly, 65,317 compounds were predicted to have an activity score of 0.75 or higher for both targets when predicting dual-target activity against AChE/MAOB. Thirdly, based on the QSAR model predicting BBB permeability, 64,619 molecules were predicted to cross the BBB. Similarly, 21,669 compounds with HIA and Caco2 scores greater than 0.75 for both bioavailability and membrane permeability were predicted to pass the QSAR models. Finally, I ranked the filtered molecules with Score 4 and selected the top-ranked molecule with a score of 3.736 (Score 4).

My scoring systems revealed the profiles of Ladostigil and the top-ranked molecule in Fig. 3C, highlighting their potential as dual inhibitors for AChE and MAOB. Firstly, Latostigil displays the QSAR values of 0.619 for AChE and 0.611 MAOB. In contrast, the top-ranked molecule exhibits QSAR values of 0.889 for AChE and 0.770 for MAOB, indicating a stronger potential for interaction with both AChE and MAOB enzymes. Additionally, Ladostigil is characterized by a RAscore of 0.994, a lipophilicity (LogP) of 2.347, a solubility (LogS) of − 2.679, BBB of 0.937, HIA of 0.987, and Caco2 of − 4.644, showing good synthesizability and favorable pharmacokinetic properties. In comparison, the top-ranked molecule has a RAscore of 0.989, LogP of 3.297, LogS of − 4.097, BBB of 0.947, HIA of 0.953, and Caco2 of − 4.976, also suggesting good synthesizability and effective pharmacokinetic profiles.

**Fig. 4:**
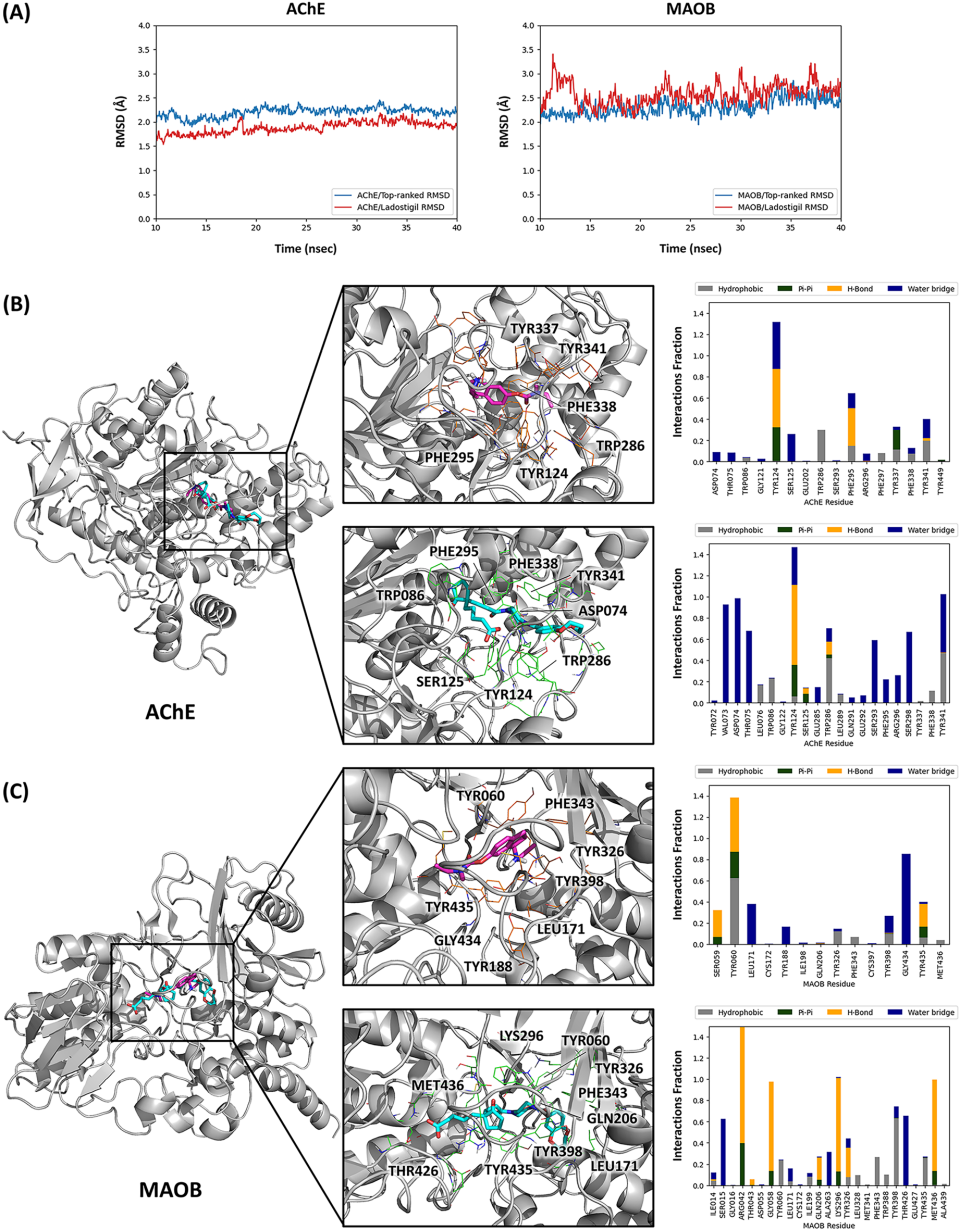
Molecular dynamics simulations for AChE/MAOB dual target **A** The trajectories in the molecular dynamics simulations are depicted in AChE and MAOB dual targets. The heavy atoms in AChE and MAOB systems with a top-ranked molecule are shown in blue, while those with Ladostigil are shown in red. **B**, **C** Key residues and important interactions observed in the molecular dynamics simulations in AChE (**B**) and MAOB (**C**) are depicted. These interactions within the protein–ligand complexes are categorized into three types: Hydrophobic, Pi-Pi, Hydrogen bonds, and Water-bridge interactions

Furthermore, I performed molecular docking and dynamics simulations to investigate the molecular interactions between the two molecules and dual targets (AChE/MAOB), which are illustrated in Fig. 4. I employed molecular docking simulations to make protein–ligand complexes for each target with this top-ranked molecule. The most favorable docking poses were selected based on the best docking scores. Ladostigil achieved docking scores of − 4.202 for AChE and − 4.891 for MAOB, while the top-ranked molecule exhibited higher docking scores of − 8.545 for AChE and − 11.059 for MAOB. Subsequent molecular dynamics simulations were performed, with their results presented in Fig. 4A. These simulations revealed that the AChE and MAOB protein–ligand complexes with Ladostigil and the top-ranked molecule exhibited fluctuations around the thermal average (1–3 Å), demonstrating the stable binding to each target throughout the simulation. The predicted poses and important key residues in the simulations are shown in Fig. 4B and C. In the AChE, Ladostigil showed interactions with Y124, W286, F295, Y337, F338, and Y341, while the top-ranked molecules had interactions with D74, W86, Y124, S125, F295, F338, and Y341. In the MAOB, Ladostigil had interactions with Y60, L171, Y188, Y326, F343, Y398, G434, and Y435, while the top-ranked molecule has interactions with Y60, L171, Q206, K296, Y326, F343, Y398, T426, Y435, and M436. Compared to Ladostigil, the top-ranked molecule showed better QSAR scores and molecular simulation results for both AChE and MAOB with similar pharmacokinetic properties in other metrics, such as LogP, LogS, BBB, HIA, and Caco2. Therefore, my SAGE methodology is effective in generating novel molecules with multiple predicted desirable properties for AChE/MAOB dual targets, attributed to my scoring-assisted generative exploration strategy with multiple QSAR models.

## Discussion

Generative deep learning provides an alternative approach to traditional methods of drug design such as virtual screening and combinatorial sampling. In this study, I developed SAGE with three functions. First, SMILES-based DNN models generate a variety of chemical structures after pre-training on pre-existing compound libraries. Selecting an initial compound library for the pre-training suitable for the task is crucial. I employed the commonly used metrics in de novo design (validity, uniqueness, novelty, and internal diversity) to compare the pre-trained models, but it’s worth noting that internal diversity cannot capture all aspects of a compound’s diversity [63]. Second, these structures are chemically diversified with mutate, crossover, and virtual synthesis operators, which allow for the generation of more complex molecules such as bridged bicyclic rings. Third, various scoring models are applied to select top-ranking molecules based on key properties necessary for drug-likeness. The SAGE was evaluated on six targets and achieved high scores of over 0.9 within an average of 10 steps for Score 1. The SAGE for Scores 2 and 3 found high-scoring molecules of over 1.9 and 2.9 within 12 steps and 14 steps on average, respectively. The SAGE was able to maximize Score 4, achieving high scores of over 3.7 within 50 steps. In my study, I segmented the steps into evaluating the pharmacodynamics and pharmacokinetics effects of the generated molecules, reflecting that addressing pharmacodynamics is prioritized before considering pharmacokinetics in drug design, rather than adhering to the principles of curriculum learning. However, the SAGE can be further advanced by employing curriculum learning based on the principle that curriculum learning can reduce complexity by breaking down complex objectives into simpler constituent objectives. As a result, SAGE generated drug-like molecules with desired properties by directing generative exploration towards high-scoring molecules, facilitating inhibitor discovery for six protein targets and even dual targets.

Muti-target drugs are gaining popularity in the fight against difficult diseases but designing them computationally is challenging. To explore new chemical entities for multi-target drugs, deep generative models can be used to generate molecules that meet the desired multi-target specificity. In this study, the SAGE was used to predict dual-target specificity for AChE and MAOB and to search for molecules predicted to be active by each QSAR model. The QSAR is a cost-effective and time-efficient method for identifying active compounds, but the reliability and accuracy of these models are reliant on the quality of the training data and limited to their application domain. The QSAR models implemented in SAGE were based on molecular fingerprints with general applicability for small molecules, which may help the successful applications in this work. However, extrapolation outside this domain may lead to reduced reliability and generative models may generate molecules outside this domain, leading to a biased exploration of chemical space. To increase the likelihood of generating compounds with desired properties using generative models, careful consideration of the application domain in the predictive models is necessary, allowing for efficient exploration of chemical space [64, 65].

Deep generative models like SAGE are revolutionizing drug discovery by enabling more efficient and cost-effective processes. By using various scoring models, SAGE identified molecules with high scores for each desired objective in a drug-like chemical space. Moreover, by defining desirable objectives with multiple scoring models, SAGE can more effectively explore chemical space through iterative fine-tuning. This breakthrough in de novo molecular design using deep learning is paving the way for more efficient and cost-effective drug discovery processes. With the ability to rapidly explore vast chemical spaces and generate novel molecules with desired properties, deep learning-based approaches like SAGE have the potential to revolutionize the field of drug discovery and development.

### Supplementary Information

**Additional file 1: Table S1.** Hyperparameter Setting for Tuning Procedure. **Table S2.** Performance Metrics of Pre-trained Models in this work. **Table S3.** Performance Metrics of QSAR Models for Target Specificity Tasks. **Table S4.** Optimal Hyperparameters of QSAR Models for Target Specificity Tasks. **Table S5.** Performance Metrics of QSAR Models for ADME/T Regression Tasks. **Table S6.** Optimal Hyperparameters of QSAR Models for ADME/T Regression Tasks. **Table S7.** Performance Metrics of QSAR Models for ADME/T Classification Tasks. **Table S8.** Optimal Hyperparameters of QSAR Models for ADME/T Classification Tasks.

## Data Availability

All results in this work can be found in the GitHub repository (github.com/hclim0213/SAGE).
