# Supplementary material for: Development of scoring-assisted generative exploration (SAGE) and its application to dual inhibitor design for acetylcholinesterase and monoamine oxidase B
Source: J Cheminform. 2024 May 24;16:59. doi: 10.1186/s13321-024-00845-w (PMC11127438; doi:10.1186/s13321-024-00845-w)
Supplement: Supplementary file 1 — Additional file 1: Table S1. Hyperparameter Setting for Tuning Procedure. Table S2. Performance Metrics of Pre-trained Models in this work. Table S3. Performance Metrics of QSAR Models for Target Specificity Tasks. Table S4. Optimal Hyperparameters of QSAR Models for Target Specificity Tasks. Table S5. Performance Metrics of QSAR Models for ADME/T Regression Tasks. Table S6. Optimal Hyperparameters of QSAR Models for ADME/T Regression Tasks. Table S7. Performance Metrics of QSAR Models for ADME/T Classification Tasks. Table S8. Optimal Hyperparameters of QSAR Models for ADME/T Classification Tasks. [file 13321_2024_845_MOESM1_ESM.pdf]

Additional Information for

Development of Scoring-Assisted Generative Exploration (SAGE) and Its Application to Dual Inhibitor Design for Acetylcholinesterase and Monoamine Oxidase B

Hocheol Lim[a,*]

[a] Bioinformatics and Molecular Design Research Center (BMDRC), Incheon, Republic of Korea

[*] Corresponding author: Hocheol Lim (ihc0213@yonsei.ac.kr)

The additional information for 'Development of Scoring-Assisted Generative Exploration (SAGE) and Its Application to Dual Inhibitor Design for Acetylcholinesterase and Monoamine Oxidase B' includes Table S1 for hyperparameter tuning procedure, Table S2 for pre-trained models, Table S3-S4 for QSAR models for target specificity tasks, Table S5-S6 for QSAR models for ADME/T regression tasks, and Table S7-S8 for QSAR models for ADME/T classification tasks.

In this study, there are many abbreviations as follows. 2D, Two dimension; AChE, Acetylcholinesterase; AD, Alzheimer's disease; ADME, Absorption, distribution, metabolism, and excretion; ADME/T, Absorption, distribution, metabolism, excretion, and toxicity; APP, Amyloid precursor protein; BBB, Blood brain barrier permeability; Caco2, Caco-2 membrane permeability; COX-2, Cyclooxygenase-2; CYP, Cytochrome P450; DILI, Drug-induced liver injury; DINGOS, Design of innovative new chemical entities generated by optimization strategies; DNN, Deep neural network; ECFP, Extended-connectivity fingerprint; FCFP, Function-class fingerprint; FGFR1, Fibroblast growth factor receptor 1; GA, Genetic algorithms; GBM, Gradient boosting machine; GEGL, Genetic expert-guided learning; hERG, Human ether-à-go-go; HIA, Human intestinal absorption; LD50, Lethal dose 50; LGBM, Light gradient boosting machine; LSTM, Long-short term memory; MACCS, Molecular access system; MAO, Monoamine oxidase; MAOB, Monoamine oxidase B; MPO, Multiple property optimization; PCFP, PubChem fingerprint; PDB, Protein data bank; Pgp, P-glycoprotein inhibition; PKCB, Protein kinase C beta; PPBR, Human plasma protein binding rate; PTP1B, Protein-tyrosine phosphatase 1B; QED, Quantitative estimate of drug-likeness; QSAR, Quantitative structure-activity relationship; QSPR, Quantitative structure-property relationship; RAscore, Retrosynthetic accessibility score; RF, Random forest; RMSD, root-mean-square deviation; SAGE, Scoring-Assisted Generative Exploration; SMILES, Simplified molecular-input line-entry system; TPSA, Topological polar surface area; XGB, Extreme gradient boosting.

Table S1. Hyperparameter Setting for Tuning Procedure

Method	Tuning parameters	Fixed parameters
GBM	n_estimators = 50, 100, 500, 1000, 1500, 2000, 2500, 3000<br>max_features = 'auto', 'sqrt', 'log2'<br>max_depth = 10, 15<br>learning_rate = 0.01, 0.05	if it is classification task,<br>class_weight = 'balanced'
LGBM	n_estimators = 50, 100, 500, 1000, 1500, 2000, 2500, 3000<br>learning_rate = 0.01, 0.05	if it is classification task,<br>class_weight = 'balanced'
RF	n_estimators = 50, 100, 500, 1000, 1500, 2000, 2500, 3000<br>max_features = 'auto', 'sqrt', 'log2'	if it is classification task,<br>class_weight = 'balanced'
XGB	n_estimators = 50, 100, 500, 1000, 1500, 2000, 2500, 3000<br>max_depth = 10, 15<br>learning_rate = 0.01, 0.05	gamma = 0<br>min_child_weight = 1<br>subsample = 0.5

Table S2. Performance Metrics of Pre-trained Models in this work

Dataset	Samples	Validity	Uniqueness	Unique_1k	Novelty	IntDiv_1	IntDiv_2
ChEMBL24	1,000	0.909	0.998	-	0.750	0.719	0.610
	3,000	0.904	0.999	0.998	0.762	0.710	0.600
	5,000	0.902	0.999	1.000	0.758	0.709	0.597
Synthetics	1,000	0.969	1.000	-	0.899	0.818	0.754
	3,000	0.963	1.000	1.000	0.898	0.808	0.737
	5,000	0.964	1.000	1.000	0.898	0.809	0.739
ZINC	1,000	0.939	1.000	-	0.996	0.769	0.675
	3,000	0.925	1.000	1.000	0.995	0.748	0.645
	5,000	0.929	1.000	1.000	0.995	0.754	0.654
ZINC-NP	1,000	0.891	1.000	-	0.497	0.688	0.573
	3,000	0.895	0.998	1.000	0.538	0.694	0.580
	5,000	0.890	0.992	1.000	0.511	0.686	0.571

Table S3. Performance Metrics of QSAR Models for Target Specificity Tasks (continue)

Task	Descriptor	Method	Train AUC	Validation AUC	Test AUC
AChE	MACCS+ECFP6	GBM	1.000 ± 0.000	0.922 ± 0.047	0.925
	MACCS+ECFP6	LGBM	0.987 ± 0.002	0.937 ± 0.035	0.931
	MACCS+ECFP6	RF	1.000 ± 0.000	0.974 ± 0.014	0.931
	MACCS+ECFP6	XGB	0.995 ± 0.001	0.948 ± 0.027	0.936
	MACCS+FCFP4	GBM	0.999 ± 0.001	0.845 ± 0.135	0.936
	MACCS+FCFP4	LGBM	0.984 ± 0.003	0.923 ± 0.050	0.941
	MACCS+FCFP4	RF	1.000 ± 0.000	0.974 ± 0.014	0.936
	MACCS+FCFP4	XGB	0.993 ± 0.001	0.948 ± 0.031	0.936
	MACCS+PCFP	GBM	1.000 ± 0.000	0.876 ± 0.051	0.947
	MACCS+PCFP	LGBM	0.978 ± 0.005	0.922 ± 0.039	0.947
	**MACCS+PCFP**	**RF**	**1.000 ± 0.000**	**0.968 ± 0.022**	**0.947**
	MACCS+PCFP	XGB	0.992 ± 0.001	0.944 ± 0.026	0.942
COX-2	MACCS+ECFP6	GBM	0.998 ± 0.001	0.697 ± 0.084	0.812
	MACCS+ECFP6	LGBM	0.927 ± 0.013	0.734 ± 0.111	0.795
	MACCS+ECFP6	RF	1.000 ± 0.000	0.780 ± 0.116	0.812
	MACCS+ECFP6	XGB	0.972 ± 0.003	0.774 ± 0.120	0.789
	MACCS+FCFP4	GBM	0.996 ± 0.003	0.716 ± 0.111	0.790
	MACCS+FCFP4	LGBM	0.936 ± 0.006	0.706 ± 0.121	0.789
	**MACCS+FCFP4**	**RF**	**1.000 ± 0.000**	**0.789 ± 0.104**	**0.818**
	MACCS+FCFP4	XGB	0.971 ± 0.001	0.766 ± 0.117	0.801
	MACCS+PCFP	GBM	0.997 ± 0.002	0.716 ± 0.134	0.795
	MACCS+PCFP	LGBM	0.937 ± 0.007	0.740 ± 0.131	0.801
	MACCS+PCFP	RF	1.000 ± 0.000	0.771 ± 0.112	0.795
	MACCS+PCFP	XGB	0.967 ± 0.002	0.777 ± 0.120	0.812
PKCB	MACCS+ECFP6	GBM	1.000 ± 0.000	0.837 ± 0.103	0.880
	MACCS+ECFP6	LGBM	0.968 ± 0.008	0.899 ± 0.097	0.882
	MACCS+ECFP6	RF	1.000 ± 0.000	0.919 ± 0.100	0.859
	MACCS+ECFP6	XGB	0.994 ± 0.002	0.897 ± 0.127	0.917
	MACCS+FCFP4	GBM	1.000 ± 0.000	0.875 ± 0.126	0.931
	MACCS+FCFP4	LGBM	0.963 ± 0.008	0.854 ± 0.134	0.896
	**MACCS+FCFP4**	**RF**	**1.000 ± 0.000**	**0.941 ± 0.070**	**0.894**
	MACCS+FCFP4	XGB	0.991 ± 0.002	0.901 ± 0.120	0.880
	MACCS+PCFP	GBM	1.000 ± 0.000	0.821 ± 0.139	0.845
	MACCS+PCFP	LGBM	0.968 ± 0.010	0.882 ± 0.110	0.861
	MACCS+PCFP	RF	1.000 ± 0.000	0.945 ± 0.065	0.861
	MACCS+PCFP	XGB	0.992 ± 0.003	0.924 ± 0.070	0.864

Table S3. Performance Metrics of QSAR Models for Target Specificity Tasks (continue)

Task	Descriptor	Method	Train AUC	Validation AUC	Test AUC
FGFR1	MACCS+ECFP6	GBM	1.000 ± 0.000	0.823 ± 0.160	0.947
	MACCS+ECFP6	LGBM	0.971 ± 0.005	0.880 ± 0.183	0.877
	**MACCS+ECFP6**	**RF**	**1.000 ± 0.000**	**0.954 ± 0.064**	**0.930**
	MACCS+ECFP6	XGB	0.989 ± 0.002	0.921 ± 0.113	0.912
	MACCS+FCFP4	GBM	1.000 ± 0.000	0.866 ± 0.153	0.947
	MACCS+FCFP4	LGBM	0.966 ± 0.006	0.893 ± 0.145	0.929
	MACCS+FCFP4	RF	1.000 ± 0.000	0.926 ± 0.076	0.930
	MACCS+FCFP4	XGB	0.986 ± 0.004	0.924 ± 0.104	0.913
	MACCS+PCFP	GBM	1.000 ± 0.000	0.885 ± 0.103	0.895
	MACCS+PCFP	LGBM	0.966 ± 0.005	0.868 ± 0.103	0.895
	MACCS+PCFP	RF	1.000 ± 0.000	0.939 ± 0.066	0.895
	MACCS+PCFP	XGB	0.985 ± 0.003	0.897 ± 0.103	0.895
PTP1B	MACCS+ECFP6	GBM	1.000 ± 0.000	0.793 ± 0.078	0.962
	MACCS+ECFP6	LGBM	0.960 ± 0.012	0.684 ± 0.223	0.907
	MACCS+ECFP6	RF	1.000 ± 0.000	0.931 ± 0.099	0.923
	MACCS+ECFP6	XGB	0.994 ± 0.002	0.841 ± 0.158	0.926
	MACCS+FCFP4	GBM	1.000 ± 0.000	0.786 ± 0.099	0.926
	MACCS+FCFP4	LGBM	0.967 ± 0.011	0.738 ± 0.195	0.926
	MACCS+FCFP4	RF	1.000 ± 0.000	0.930 ± 0.070	0.945
	MACCS+FCFP4	XGB	0.991 ± 0.002	0.847 ± 0.160	0.942
	MACCS+PCFP	GBM	1.000 ± 0.000	0.919 ± 0.078	0.849
	MACCS+PCFP	LGBM	0.977 ± 0.009	0.829 ± 0.172	0.868
	**MACCS+PCFP**	**RF**	**1.000 ± 0.000**	**0.944 ± 0.069**	**0.947**
	MACCS+PCFP	XGB	0.994 ± 0.002	0.907 ± 0.105	0.885
MAOB	MACCS+ECFP6	GBM	1.000 ± 0.000	0.752 ± 0.128	0.842
	MACCS+ECFP6	LGBM	0.943 ± 0.008	0.780 ± 0.182	0.783
	**MACCS+ECFP6**	**RF**	**1.000 ± 0.000**	**0.901 ± 0.085**	**0.842**
	MACCS+ECFP6	XGB	0.983 ± 0.003	0.846 ± 0.123	0.823
	MACCS+FCFP4	GBM	1.000 ± 0.000	0.732 ± 0.163	0.822
	MACCS+FCFP4	LGBM	0.946 ± 0.014	0.809 ± 0.135	0.803
	MACCS+FCFP4	RF	1.000 ± 0.000	0.885 ± 0.104	0.842
	MACCS+FCFP4	XGB	0.977 ± 0.003	0.852 ± 0.129	0.823
	MACCS+PCFP	GBM	1.000 ± 0.000	0.790 ± 0.120	0.822
	MACCS+PCFP	LGBM	0.941 ± 0.012	0.802 ± 0.103	0.725
	MACCS+PCFP	RF	1.000 ± 0.000	0.881 ± 0.106	0.822
	MACCS+PCFP	XGB	0.975 ± 0.003	0.848 ± 0.100	0.784

Table S4. Optimal Hyperparameters of QSAR Models for Target Specificity Tasks (continue)

Task	Descriptor	Method	Optimal Hyperarameters
AChE	MACCS+ECFP6	GBM	{'learning_rate': 0.01, 'max_depth': 15, 'max_features': 'log2', 'n_estimators': 1000}
	MACCS+ECFP6	LGBM	{'learning_rate': 0.01, 'n_estimators': 3000}
	MACCS+ECFP6	RF	{'max_features': 'log2', 'n_estimators': 3000}
	MACCS+ECFP6	XGB	{'learning_rate': 0.01, 'max_depth': 10, 'n_estimators': 2500}
	MACCS+FCFP4	GBM	{'learning_rate': 0.01, 'max_depth': 15, 'max_features': 'log2', 'n_estimators': 1000}
	MACCS+FCFP4	LGBM	{'learning_rate': 0.05, 'n_estimators': 2500}
	MACCS+FCFP4	RF	{'max_features': 'log2', 'n_estimators': 1000}
	MACCS+FCFP4	XGB	{'learning_rate': 0.01, 'max_depth': 10, 'n_estimators': 1500}
	MACCS+PCFP	GBM	{'learning_rate': 0.01, 'max_depth': 15, 'max_features': 'sqrt', 'n_estimators': 100}
	MACCS+PCFP	LGBM	{'learning_rate': 0.05, 'n_estimators': 500}
	**MACCS+PCFP**	**RF**	**{'max_features': 'log2', 'n_estimators': 500}**
	MACCS+PCFP	XGB	{'learning_rate': 0.01, 'max_depth': 15, 'n_estimators': 1500}
COX-2	MACCS+ECFP6	GBM	{'learning_rate': 0.01, 'max_depth': 15, 'max_features': 'log2', 'n_estimators': 100}
	MACCS+ECFP6	LGBM	{'learning_rate': 0.01, 'n_estimators': 500}
	MACCS+ECFP6	RF	{'max_features': 'log2', 'n_estimators': 1500}
	MACCS+ECFP6	XGB	{'learning_rate': 0.05, 'max_depth': 10, 'n_estimators': 500}
	MACCS+FCFP4	GBM	{'learning_rate': 0.05, 'max_depth': 15, 'max_features': 'log2', 'n_estimators': 50}
	MACCS+FCFP4	LGBM	{'learning_rate': 0.01, 'n_estimators': 1000}
	**MACCS+FCFP4**	**RF**	**{'max_features': 'auto', 'n_estimators': 100}**
	MACCS+FCFP4	XGB	{'learning_rate': 0.05, 'max_depth': 15, 'n_estimators': 100}
	MACCS+PCFP	GBM	{'learning_rate': 0.05, 'max_depth': 10, 'max_features': 'log2', 'n_estimators': 100}
	MACCS+PCFP	LGBM	{'learning_rate': 0.01, 'n_estimators': 1000}
	MACCS+PCFP	RF	{'max_features': 'log2', 'n_estimators': 500}
	MACCS+PCFP	XGB	{'learning_rate': 0.01, 'max_depth': 10, 'n_estimators': 500}
PKCB	MACCS+ECFP6	GBM	{'learning_rate': 0.05, 'max_depth': 15, 'max_features': 'log2', 'n_estimators': 500}
	MACCS+ECFP6	LGBM	{'learning_rate': 0.01, 'n_estimators': 2500}
	MACCS+ECFP6	RF	{'max_features': 'log2', 'n_estimators': 100}
	MACCS+ECFP6	XGB	{'learning_rate': 0.05, 'max_depth': 10, 'n_estimators': 500}
	MACCS+FCFP4	GBM	{'learning_rate': 0.05, 'max_depth': 10, 'max_features': 'log2', 'n_estimators': 500}
	MACCS+FCFP4	LGBM	{'learning_rate': 0.01, 'n_estimators': 1500}
	**MACCS+FCFP4**	**RF**	**{'max_features': 'log2', 'n_estimators': 3000}**
	MACCS+FCFP4	XGB	{'learning_rate': 0.05, 'max_depth': 10, 'n_estimators': 2500}
	MACCS+PCFP	GBM	{'learning_rate': 0.01, 'max_depth': 10, 'max_features': 'log2', 'n_estimators': 1000}
	MACCS+PCFP	LGBM	{'learning_rate': 0.05, 'n_estimators': 2500}
	MACCS+PCFP	RF	{'max_features': 'log2', 'n_estimators': 2000}
	MACCS+PCFP	XGB	{'learning_rate': 0.01, 'max_depth': 10, 'n_estimators': 2500}

Table S4. Optimal Hyperparameters of QSAR Models for Target Specificity Tasks (continue)

Task	Descriptor	Method	Optimal Hyperarameters
FGFR1	MACCS+ECFP6	GBM	{'learning_rate': 0.05, 'max_depth': 10, 'max_features': 'log2', 'n_estimators': 100}
	MACCS+ECFP6	LGBM	{'learning_rate': 0.05, 'n_estimators': 50}
	**MACCS+ECFP6**	**RF**	**{'max_features': 'auto', 'n_estimators': 50}**
	MACCS+ECFP6	XGB	{'learning_rate': 0.05, 'max_depth': 10, 'n_estimators': 100}
	MACCS+FCFP4	GBM	{'learning_rate': 0.01, 'max_depth': 15, 'max_features': 'log2', 'n_estimators': 100}
	MACCS+FCFP4	LGBM	{'learning_rate': 0.01, 'n_estimators': 2500}
	MACCS+FCFP4	RF	{'max_features': 'log2', 'n_estimators': 2500}
	MACCS+FCFP4	XGB	{'learning_rate': 0.05, 'max_depth': 10, 'n_estimators': 50}
	MACCS+PCFP	GBM	{'learning_rate': 0.05, 'max_depth': 10, 'max_features': 'sqrt', 'n_estimators': 50}
	MACCS+PCFP	LGBM	{'learning_rate': 0.01, 'n_estimators': 3000}
	MACCS+PCFP	RF	{'max_features': 'auto', 'n_estimators': 50}
	MACCS+PCFP	XGB	{'learning_rate': 0.01, 'max_depth': 10, 'n_estimators': 1000}
PTP1B	MACCS+ECFP6	GBM	{'learning_rate': 0.05, 'max_depth': 15, 'max_features': 'sqrt', 'n_estimators': 50}
	MACCS+ECFP6	LGBM	{'learning_rate': 0.01, 'n_estimators': 3000}
	MACCS+ECFP6	RF	{'max_features': 'log2', 'n_estimators': 100}
	MACCS+ECFP6	XGB	{'learning_rate': 0.01, 'max_depth': 10, 'n_estimators': 3000}
	MACCS+FCFP4	GBM	{'learning_rate': 0.05, 'max_depth': 15, 'max_features': 'log2', 'n_estimators': 3000}
	MACCS+FCFP4	LGBM	{'learning_rate': 0.05, 'n_estimators': 500}
	MACCS+FCFP4	RF	{'max_features': 'log2', 'n_estimators': 50}
	MACCS+FCFP4	XGB	{'learning_rate': 0.01, 'max_depth': 10, 'n_estimators': 2500}
	MACCS+PCFP	GBM	{'learning_rate': 0.05, 'max_depth': 10, 'max_features': 'auto', 'n_estimators': 3000}
	MACCS+PCFP	LGBM	{'learning_rate': 0.01, 'n_estimators': 1000}
	**MACCS+PCFP**	**RF**	**{'max_features': 'auto', 'n_estimators': 1000}**
	MACCS+PCFP	XGB	{'learning_rate': 0.01, 'max_depth': 10, 'n_estimators': 1000}
MAOB	MACCS+ECFP6	GBM	{'learning_rate': 0.01, 'max_depth': 10, 'max_features': 'log2', 'n_estimators': 50}
	MACCS+ECFP6	LGBM	{'learning_rate': 0.01, 'n_estimators': 1000}
	**MACCS+ECFP6**	**RF**	**{'max_features': 'log2', 'n_estimators': 100}**
	MACCS+ECFP6	XGB	{'learning_rate': 0.01, 'max_depth': 10, 'n_estimators': 1000}
	MACCS+FCFP4	GBM	{'learning_rate': 0.01, 'max_depth': 15, 'max_features': 'log2', 'n_estimators': 50}
	MACCS+FCFP4	LGBM	{'learning_rate': 0.05, 'n_estimators': 100}
	MACCS+FCFP4	RF	{'max_features': 'log2', 'n_estimators': 2500}
	MACCS+FCFP4	XGB	{'learning_rate': 0.01, 'max_depth': 10, 'n_estimators': 2500}
	MACCS+PCFP	GBM	{'learning_rate': 0.01, 'max_depth': 15, 'max_features': 'log2', 'n_estimators': 50}
	MACCS+PCFP	LGBM	{'learning_rate': 0.05, 'n_estimators': 100}
	MACCS+PCFP	RF	{'max_features': 'auto', 'n_estimators': 1500}
	MACCS+PCFP	XGB	{'learning_rate': 0.05, 'max_depth': 10, 'n_estimators': 100}

Table S5. Performance Metrics of QSAR Models for ADME/T Regression Tasks

Task	Descriptor	Method	Train MAE	Validation MAE	Test MAE
Caco2	MACCS+ECFP6	GBM	0.419 ± 0.011	0.538 ± 0.078	0.405
	MACCS+ECFP6	LGBM	0.490 ± 0.008	0.538 ± 0.080	0.387
	MACCS+ECFP6	RF	0.130 ± 0.001	0.414 ± 0.052	0.383
	MACCS+ECFP6	XGB	3.471 ± 0.007	3.471 ± 0.087	0.352
	MACCS+FCFP4	GBM	0.415 ± 0.007	0.502 ± 0.082	0.335
	MACCS+FCFP4	LGBM	0.480 ± 0.006	0.521 ± 0.079	0.336
	**MACCS+FCFP4**	**RF**	**0.125 ± 0.002**	**0.391 ± 0.047**	**0.348**
	MACCS+FCFP4	XGB	3.471 ± 0.008	3.471 ± 0.085	0.325
	MACCS+PCFP	GBM	0.411 ± 0.009	0.529 ± 0.072	0.316
	MACCS+PCFP	LGBM	0.481 ± 0.008	0.527 ± 0.081	0.325
	MACCS+PCFP	RF	0.125 ± 0.002	0.410 ± 0.056	0.339
	MACCS+PCFP	XGB	3.471 ± 0.007	3.471 ± 0.086	0.305
PPBR	MACCS+ECFP6	GBM	10.014 ± 0.232	12.391 ± 1.356	9.387
	MACCS+ECFP6	LGBM	11.278 ± 0.208	12.266 ± 1.1	9.718
	MACCS+ECFP6	RF	3.781 ± 0.080	11.116 ± 1.279	9.760
	MACCS+ECFP6	XGB	52.386 ± 0.157	52.809 ± 1.87	9.749
	MACCS+FCFP4	GBM	10.478 ± 0.188	12.297 ± 1.22	9.296
	MACCS+FCFP4	LGBM	11.325 ± 0.211	12.304 ± 1.199	8.746
	**MACCS+FCFP4**	**RF**	**3.736 ± 0.088**	**10.926 ± 1.414**	**9.126**
	MACCS+FCFP4	XGB	52.332 ± 0.148	52.722 ± 1.979	8.974
	MACCS+PCFP	GBM	9.611 ± 0.191	12.139 ± 1.405	9.191
	MACCS+PCFP	LGBM	11.255 ± 0.219	12.283 ± 1.278	8.317
	MACCS+PCFP	RF	3.748 ± 0.086	10.940 ± 1.484	9.470
	MACCS+PCFP	XGB	52.397 ± 0.153	52.747 ± 1.838	8.989
LD50	MACCS+ECFP6	GBM	0.541 ± 0.004	0.600 ± 0.052	0.605
	MACCS+ECFP6	LGBM	0.594 ± 0.006	0.612 ± 0.055	0.626
	MACCS+ECFP6	RF	0.161 ± 0.001	0.467 ± 0.047	0.611
	MACCS+ECFP6	XGB	1.218 ± 0.009	1.227 ± 0.093	0.599
	MACCS+FCFP4	GBM	0.541 ± 0.004	0.596 ± 0.05	0.568
	MACCS+FCFP4	LGBM	0.594 ± 0.005	0.611 ± 0.055	0.581
	MACCS+FCFP4	RF	0.158 ± 0.002	0.459 ± 0.048	0.585
	MACCS+FCFP4	XGB	1.218 ± 0.009	1.227 ± 0.094	0.581
	MACCS+PCFP	GBM	0.533 ± 0.004	0.592 ± 0.051	0.565
	MACCS+PCFP	LGBM	0.592 ± 0.005	0.613 ± 0.055	0.578
	**MACCS+PCFP**	**RF**	**0.154 ± 0.001**	**0.450 ± 0.047**	**0.575**
	MACCS+PCFP	XGB	1.223 ± 0.009	1.236 ± 0.096	0.574

Table S6. Optimal Hyperparameters of QSAR Models for ADME/T Regression Tasks

Task	Descriptor	Method	Optimal Hyperarameters
Caco2	MACCS+ECFP6	GBM	{'learning_rate': 0.01, 'max_depth': 10, 'max_features': 'sqrt', 'n_estimators': 1500}
	MACCS+ECFP6	LGBM	{'learning_rate': 0.01, 'n_estimators': 1000}
	MACCS+ECFP6	RF	{'max_features': 'auto', 'n_estimators': 1000}
	MACCS+ECFP6	XGB	{'learning_rate': 0.01, 'max_depth': 10, 'n_estimators': 1500}
	MACCS+FCFP4	GBM	{'learning_rate': 0.01, 'max_depth': 10, 'max_features': 'sqrt', 'n_estimators': 1500}
	MACCS+FCFP4	LGBM	{'learning_rate': 0.05, 'n_estimators': 500}
	**MACCS+FCFP4**	**RF**	**{'max_features': 'auto', 'n_estimators': 1500}**
	MACCS+FCFP4	XGB	{'learning_rate': 0.01, 'max_depth': 10, 'n_estimators': 1000}
	MACCS+PCFP	GBM	{'learning_rate': 0.01, 'max_depth': 10, 'max_features': 'sqrt', 'n_estimators': 2500}
	MACCS+PCFP	LGBM	{'learning_rate': 0.01, 'n_estimators': 1500}
	MACCS+PCFP	RF	{'max_features': 'auto', 'n_estimators': 2000}
	MACCS+PCFP	XGB	{'learning_rate': 0.01, 'max_depth': 10, 'n_estimators': 3000}
PPBR	MACCS+ECFP6	GBM	{'learning_rate': 0.05, 'max_depth': 10, 'max_features': 'auto', 'n_estimators': 1000}
	MACCS+ECFP6	LGBM	{'learning_rate': 0.01, 'n_estimators': 500}
	MACCS+ECFP6	RF	{'max_features': 'auto', 'n_estimators': 2500}
	MACCS+ECFP6	XGB	{'learning_rate': 0.01, 'max_depth': 10, 'n_estimators': 3000}
	MACCS+FCFP4	GBM	{'learning_rate': 0.01, 'max_depth': 10, 'max_features': 'auto', 'n_estimators': 1500}
	MACCS+FCFP4	LGBM	{'learning_rate': 0.01, 'n_estimators': 1000}
	**MACCS+FCFP4**	**RF**	**{'max_features': 'auto', 'n_estimators': 500}**
	MACCS+FCFP4	XGB	{'learning_rate': 0.01, 'max_depth': 10, 'n_estimators': 2000}
	MACCS+PCFP	GBM	{'learning_rate': 0.05, 'max_depth': 10, 'max_features': 'sqrt', 'n_estimators': 500}
	MACCS+PCFP	LGBM	{'learning_rate': 0.01, 'n_estimators': 1000}
	MACCS+PCFP	RF	{'max_features': 'auto', 'n_estimators': 2000}
	MACCS+PCFP	XGB	{'learning_rate': 0.01, 'max_depth': 10, 'n_estimators': 1500}
LD50	MACCS+ECFP6	GBM	{'learning_rate': 0.01, 'max_depth': 15, 'max_features': 'sqrt', 'n_estimators': 3000}
	MACCS+ECFP6	LGBM	{'learning_rate': 0.01, 'n_estimators': 2500}
	MACCS+ECFP6	RF	{'max_features': 'auto', 'n_estimators': 3000}
	MACCS+ECFP6	XGB	{'learning_rate': 0.01, 'max_depth': 15, 'n_estimators': 2500}
	MACCS+FCFP4	GBM	{'learning_rate': 0.01, 'max_depth': 15, 'max_features': 'sqrt', 'n_estimators': 2500}
	MACCS+FCFP4	LGBM	{'learning_rate': 0.01, 'n_estimators': 3000}
	MACCS+FCFP4	RF	{'max_features': 'auto', 'n_estimators': 3000}
	MACCS+FCFP4	XGB	{'learning_rate': 0.01, 'max_depth': 15, 'n_estimators': 2000}
	MACCS+PCFP	GBM	{'learning_rate': 0.01, 'max_depth': 10, 'max_features': 'sqrt', 'n_estimators': 3000}
	MACCS+PCFP	LGBM	{'learning_rate': 0.05, 'n_estimators': 1000}
	**MACCS+PCFP**	**RF**	**{'max_features': 'auto', 'n_estimators': 2000}**
	MACCS+PCFP	XGB	{'learning_rate': 0.01, 'max_depth': 10, 'n_estimators': 2000}

Table S7. Performance Metrics of QSAR Models for ADME/T Classification Tasks (continue)

Task	Descriptor	Method	Train AUC	Validation AUC	Test AUC
HIA	MACCS+ECFP6	GBM	1.000 ± 0.000	0.876 ± 0.116	0.611
	MACCS+ECFP6	LGBM	0.976 ± 0.005	0.853 ± 0.094	0.915
	MACCS+ECFP6	RF	1.000 ± 0.000	0.929 ± 0.067	0.667
	MACCS+ECFP6	XGB	0.994 ± 0.001	0.886 ± 0.097	0.852
	MACCS+FCFP4	GBM	0.999 ± 0.003	0.846 ± 0.151	0.722
	MACCS+FCFP4	LGBM	0.969 ± 0.009	0.888 ± 0.058	0.878
	MACCS+FCFP4	RF	1.000 ± 0.000	0.966 ± 0.035	0.778
	MACCS+FCFP4	XGB	0.991 ± 0.001	0.915 ± 0.089	0.796
	MACCS+PCFP	GBM	1.000 ± 0.001	0.915 ± 0.081	0.611
	MACCS+PCFP	LGBM	0.974 ± 0.006	0.871 ± 0.081	0.939
	MACCS+PCFP	RF	1.000 ± 0.000	0.927 ± 0.062	0.648
	**MACCS+PCFP**	**XGB**	**0.988 ± 0.002**	**0.935 ± 0.055**	**0.889**
Pgp	MACCS+ECFP6	GBM	1.000 ± 0.000	0.829 ± 0.082	0.858
	MACCS+ECFP6	LGBM	0.970 ± 0.003	0.869 ± 0.080	0.833
	MACCS+ECFP6	RF	1.000 ± 0.000	0.909 ± 0.037	0.858
	MACCS+ECFP6	XGB	0.987 ± 0.001	0.903 ± 0.060	0.849
	MACCS+FCFP4	GBM	0.998 ± 0.001	0.832 ± 0.078	0.849
	MACCS+FCFP4	LGBM	0.966 ± 0.003	0.872 ± 0.096	0.858
	**MACCS+FCFP4**	**RF**	**1.000 ± 0.000**	**0.900 ± 0.049**	**0.874**
	MACCS+FCFP4	XGB	0.983 ± 0.002	0.899 ± 0.072	0.862
	MACCS+PCFP	GBM	0.999 ± 0.000	0.833 ± 0.079	0.866
	MACCS+PCFP	LGBM	0.965 ± 0.003	0.871 ± 0.092	0.797
	MACCS+PCFP	RF	1.000 ± 0.000	0.895 ± 0.070	0.866
	MACCS+PCFP	XGB	0.981 ± 0.001	0.892 ± 0.078	0.874
BBB	MACCS+ECFP6	GBM	0.996 ± 0.002	0.820 ± 0.098	0.749
	MACCS+ECFP6	LGBM	0.963 ± 0.002	0.871 ± 0.048	0.787
	MACCS+ECFP6	RF	1.000 ± 0.000	0.885 ± 0.056	0.754
	MACCS+ECFP6	XGB	0.980 ± 0.002	0.868 ± 0.051	0.777
	MACCS+FCFP4	GBM	0.981 ± 0.007	0.794 ± 0.059	0.775
	MACCS+FCFP4	LGBM	0.959 ± 0.003	0.855 ± 0.043	0.78
	MACCS+FCFP4	RF	1.000 ± 0.000	0.893 ± 0.035	0.766
	**MACCS+FCFP4**	**XGB**	**0.967 ± 0.002**	**0.878 ± 0.041**	**0.807**
	MACCS+PCFP	GBM	0.995 ± 0.003	0.801 ± 0.071	0.788
	MACCS+PCFP	LGBM	0.960 ± 0.004	0.845 ± 0.073	0.807
	MACCS+PCFP	RF	1.000 ± 0.000	0.892 ± 0.044	0.778
	MACCS+PCFP	XGB	0.972 ± 0.002	0.885 ± 0.057	0.776

Table S7. Performance Metrics of QSAR Models for ADME/T Classification Tasks (continue)

Task	Descriptor	Method	Train AUC	Validation AUC	Test AUC
CYP2D6	MACCS+ECFP6	GBM	0.962 ± 0.006	0.784 ± 0.022	0.697
	MACCS+ECFP6	LGBM	0.852 ± 0.002	0.800 ± 0.016	0.779
	MACCS+ECFP6	RF	1.000 ± 0.000	0.842 ± 0.016	0.629
	MACCS+ECFP6	XGB	0.945 ± 0.001	0.827 ± 0.013	0.709
	MACCS+FCFP4	GBM	0.942 ± 0.003	0.808 ± 0.022	0.711
	**MACCS+FCFP4**	**LGBM**	**0.858 ± 0.002**	**0.820 ± 0.016**	**0.795**
	MACCS+FCFP4	RF	1.000 ± 0.000	0.847 ± 0.018	0.684
	MACCS+FCFP4	XGB	0.913 ± 0.002	0.830 ± 0.014	0.731
	MACCS+PCFP	GBM	0.969 ± 0.002	0.799 ± 0.014	0.703
	MACCS+PCFP	LGBM	0.853 ± 0.003	0.802 ± 0.015	0.791
	MACCS+PCFP	RF	1.000 ± 0.000	0.833 ± 0.019	0.677
	MACCS+PCFP	XGB	0.938 ± 0.002	0.833 ± 0.015	0.725
CYP3A4	MACCS+ECFP6	GBM	0.953 ± 0.003	0.823 ± 0.033	0.796
	MACCS+ECFP6	LGBM	0.860 ± 0.002	0.828 ± 0.038	0.794
	MACCS+ECFP6	RF	1.000 ± 0.000	0.864 ± 0.022	0.775
	MACCS+ECFP6	XGB	0.929 ± 0.002	0.849 ± 0.030	0.801
	MACCS+FCFP4	GBM	0.946 ± 0.003	0.827 ± 0.027	0.805
	MACCS+FCFP4	LGBM	0.863 ± 0.003	0.833 ± 0.038	0.800
	MACCS+FCFP4	RF	1.000 ± 0.000	0.859 ± 0.027	0.776
	**MACCS+FCFP4**	**XGB**	**0.925 ± 0.002**	**0.854 ± 0.030**	**0.811**
	MACCS+PCFP	GBM	0.964 ± 0.002	0.820 ± 0.025	0.781
	MACCS+PCFP	LGBM	0.863 ± 0.003	0.830 ± 0.038	0.788
	MACCS+PCFP	RF	1.000 ± 0.000	0.847 ± 0.026	0.758
	MACCS+PCFP	XGB	0.943 ± 0.002	0.856 ± 0.030	0.790
CYP2C9	MACCS+ECFP6	GBM	0.957 ± 0.002	0.808 ± 0.016	0.781
	MACCS+ECFP6	LGBM	0.853 ± 0.002	0.811 ± 0.017	0.808
	MACCS+ECFP6	RF	1.000 ± 0.000	0.857 ± 0.011	0.711
	MACCS+ECFP6	XGB	0.931 ± 0.002	0.840 ± 0.012	0.781
	MACCS+FCFP4	GBM	0.932 ± 0.003	0.813 ± 0.018	0.772
	MACCS+FCFP4	LGBM	0.849 ± 0.004	0.818 ± 0.019	0.799
	MACCS+FCFP4	RF	1.000 ± 0.000	0.859 ± 0.014	0.758
	MACCS+FCFP4	XGB	0.913 ± 0.003	0.842 ± 0.012	0.787
	MACCS+PCFP	GBM	0.960 ± 0.003	0.830 ± 0.014	0.760
	**MACCS+PCFP**	**LGBM**	**0.865 ± 0.002**	**0.834 ± 0.014**	**0.795**
	MACCS+PCFP	RF	1.000 ± 0.000	0.853 ± 0.015	0.747
	MACCS+PCFP	XGB	0.937 ± 0.001	0.858 ± 0.012	0.765

Table S7. Performance Metrics of QSAR Models for ADME/T Classification Tasks (continue)

Task	Descriptor	Method	Train AUC	Validation AUC	Test AUC
hERG	MACCS+ECFP6	GBM	0.997 ± 0.003	0.676 ± 0.102	0.669
	MACCS+ECFP6	LGBM	0.942 ± 0.008	0.780 ± 0.071	0.668
	MACCS+ECFP6	RF	1.000 ± 0.000	0.823 ± 0.057	0.67
	MACCS+ECFP6	XGB	0.972 ± 0.002	0.807 ± 0.064	0.713
	MACCS+FCFP4	GBM	0.995 ± 0.004	0.732 ± 0.075	0.708
	MACCS+FCFP4	LGBM	0.940 ± 0.008	0.810 ± 0.081	0.718
	**MACCS+FCFP4**	**RF**	**1.000 ± 0.000**	**0.820 ± 0.069**	**0.717**
	MACCS+FCFP4	XGB	0.967 ± 0.003	0.832 ± 0.060	0.699
	MACCS+PCFP	GBM	0.998 ± 0.002	0.739 ± 0.086	0.694
	MACCS+PCFP	LGBM	0.945 ± 0.004	0.781 ± 0.113	0.679
	MACCS+PCFP	RF	1.000 ± 0.000	0.817 ± 0.062	0.651
	MACCS+PCFP	XGB	0.962 ± 0.002	0.835 ± 0.088	0.69
AMES	MACCS+ECFP6	GBM	0.963 ± 0.006	0.775 ± 0.037	0.778
	MACCS+ECFP6	LGBM	0.874 ± 0.003	0.784 ± 0.048	0.761
	MACCS+ECFP6	RF	1.000 ± 0.000	0.819 ± 0.052	0.777
	MACCS+ECFP6	XGB	0.934 ± 0.003	0.808 ± 0.045	0.776
	MACCS+FCFP4	GBM	0.953 ± 0.003	0.773 ± 0.053	0.758
	MACCS+FCFP4	LGBM	0.875 ± 0.002	0.796 ± 0.044	0.758
	**MACCS+FCFP4**	**RF**	**1.000 ± 0.000**	**0.822 ± 0.064**	**0.776**
	MACCS+FCFP4	XGB	0.929 ± 0.002	0.823 ± 0.046	0.771
	MACCS+PCFP	GBM	0.954 ± 0.005	0.791 ± 0.043	0.768
	MACCS+PCFP	LGBM	0.880 ± 0.004	0.796 ± 0.039	0.774
	MACCS+PCFP	RF	1.000 ± 0.000	0.817 ± 0.068	0.772
	MACCS+PCFP	XGB	0.931 ± 0.003	0.820 ± 0.046	0.77
DILI	MACCS+ECFP6	GBM	1.000 ± 0.000	0.719 ± 0.098	0.808
	MACCS+ECFP6	LGBM	0.938 ± 0.005	0.807 ± 0.098	0.789
	MACCS+ECFP6	RF	1.000 ± 0.000	0.868 ± 0.074	0.796
	MACCS+ECFP6	XGB	0.969 ± 0.004	0.859 ± 0.086	0.809
	MACCS+FCFP4	GBM	1.000 ± 0.000	0.734 ± 0.076	0.833
	MACCS+FCFP4	LGBM	0.944 ± 0.006	0.812 ± 0.092	0.843
	MACCS+FCFP4	RF	1.000 ± 0.000	0.862 ± 0.071	0.841
	**MACCS+FCFP4**	**XGB**	**0.965 ± 0.004**	**0.854 ± 0.082**	**0.861**
	MACCS+PCFP	GBM	1.000 ± 0.001	0.721 ± 0.113	0.766
	MACCS+PCFP	LGBM	0.941 ± 0.007	0.783 ± 0.100	0.787
	MACCS+PCFP	RF	1.000 ± 0.000	0.841 ± 0.095	0.787
	MACCS+PCFP	XGB	0.966 ± 0.004	0.846 ± 0.082	0.799

Table S8. Optimal Hyperparameters of QSAR Models for ADME/T Classification Tasks (continue)

Task	Descriptor	Method	Optimal Hyperarameters
HIA	MACCS+ECFP6	GBM	{'learning_rate': 0.01, 'max_depth': 10, 'max_features': 'log2', 'n_estimators': 1000}
	MACCS+ECFP6	LGBM	{'learning_rate': 0.01, 'n_estimators': 2000}
	MACCS+ECFP6	RF	{'max_features': 'auto', 'n_estimators': 500}
	MACCS+ECFP6	XGB	{'learning_rate': 0.01, 'max_depth': 10, 'n_estimators': 1000}
	MACCS+FCFP4	GBM	{'learning_rate': 0.05, 'max_depth': 15, 'max_features': 'log2', 'n_estimators': 100}
	MACCS+FCFP4	LGBM	{'learning_rate': 0.01, 'n_estimators': 500}
	MACCS+FCFP4	RF	{'max_features': 'auto', 'n_estimators': 50}
	MACCS+FCFP4	XGB	{'learning_rate': 0.01, 'max_depth': 10, 'n_estimators': 500}
	MACCS+PCFP	GBM	{'learning_rate': 0.01, 'max_depth': 10, 'max_features': 'sqrt', 'n_estimators': 100}
	MACCS+PCFP	LGBM	{'learning_rate': 0.01, 'n_estimators': 500}
	MACCS+PCFP	RF	{'max_features': 'auto', 'n_estimators': 1000}
	**MACCS+PCFP**	**XGB**	**{'learning_rate': 0.01, 'max_depth': 10, 'n_estimators': 500}**
Pgp	MACCS+ECFP6	GBM	{'learning_rate': 0.01, 'max_depth': 15, 'max_features': 'sqrt', 'n_estimators': 500}
	MACCS+ECFP6	LGBM	{'learning_rate': 0.01, 'n_estimators': 500}
	MACCS+ECFP6	RF	{'max_features': 'auto', 'n_estimators': 1000}
	MACCS+ECFP6	XGB	{'learning_rate': 0.05, 'max_depth': 10, 'n_estimators': 100}
	MACCS+FCFP4	GBM	{'learning_rate': 0.01, 'max_depth': 10, 'max_features': 'sqrt', 'n_estimators': 1000}
	MACCS+FCFP4	LGBM	{'learning_rate': 0.01, 'n_estimators': 500}
	**MACCS+FCFP4**	**RF**	**{'max_features': 'auto', 'n_estimators': 3000}**
	MACCS+FCFP4	XGB	{'learning_rate': 0.01, 'max_depth': 15, 'n_estimators': 500}
	MACCS+PCFP	GBM	{'learning_rate': 0.05, 'max_depth': 10, 'max_features': 'sqrt', 'n_estimators': 100}
	MACCS+PCFP	LGBM	{'learning_rate': 0.01, 'n_estimators': 500}
	MACCS+PCFP	RF	{'max_features': 'auto', 'n_estimators': 500}
	MACCS+PCFP	XGB	{'learning_rate': 0.05, 'max_depth': 15, 'n_estimators': 1000}
BBB	MACCS+ECFP6	GBM	{'learning_rate': 0.01, 'max_depth': 10, 'max_features': 'sqrt', 'n_estimators': 500}
	MACCS+ECFP6	LGBM	{'learning_rate': 0.05, 'n_estimators': 1500}
	MACCS+ECFP6	RF	{'max_features': 'auto', 'n_estimators': 3000}
	MACCS+ECFP6	XGB	{'learning_rate': 0.01, 'max_depth': 10, 'n_estimators': 1000}
	MACCS+FCFP4	GBM	{'learning_rate': 0.01, 'max_depth': 10, 'max_features': 'log2', 'n_estimators': 1000}
	MACCS+FCFP4	LGBM	{'learning_rate': 0.01, 'n_estimators': 500}
	MACCS+FCFP4	RF	{'max_features': 'log2', 'n_estimators': 2000}
	**MACCS+FCFP4**	**XGB**	**{'learning_rate': 0.01, 'max_depth': 15, 'n_estimators': 1000}**
	MACCS+PCFP	GBM	{'learning_rate': 0.01, 'max_depth': 10, 'max_features': 'sqrt', 'n_estimators': 2500}
	MACCS+PCFP	LGBM	{'learning_rate': 0.01, 'n_estimators': 500}
	MACCS+PCFP	RF	{'max_features': 'auto', 'n_estimators': 2500}
	MACCS+PCFP	XGB	{'learning_rate': 0.05, 'max_depth': 10, 'n_estimators': 100}

Table S8. Optimal Hyperparameters of QSAR Models for ADME/T Classification Tasks (continue)

Task	Descriptor	Method	Optimal Hyperarameters
CYP2D6	MACCS+ECFP6	GBM	{'learning_rate': 0.01, 'max_depth': 10, 'max_features': 'sqrt', 'n_estimators': 2000}
	MACCS+ECFP6	LGBM	{'learning_rate': 0.01, 'n_estimators': 1500}
	MACCS+ECFP6	RF	{'max_features': 'log2', 'n_estimators': 2000}
	MACCS+ECFP6	XGB	{'learning_rate': 0.01, 'max_depth': 15, 'n_estimators': 1500}
	MACCS+FCFP4	GBM	{'learning_rate': 0.01, 'max_depth': 10, 'max_features': 'sqrt', 'n_estimators': 1500}
	**MACCS+FCFP4**	**LGBM**	**{'learning_rate': 0.01, 'n_estimators': 1500}**
	MACCS+FCFP4	RF	{'max_features': 'auto', 'n_estimators': 3000}
	MACCS+FCFP4	XGB	{'learning_rate': 0.01, 'max_depth': 10, 'n_estimators': 1500}
	MACCS+PCFP	GBM	{'learning_rate': 0.01, 'max_depth': 10, 'max_features': 'sqrt', 'n_estimators': 1000}
	MACCS+PCFP	LGBM	{'learning_rate': 0.01, 'n_estimators': 1500}
	MACCS+PCFP	RF	{'max_features': 'auto', 'n_estimators': 3000}
	MACCS+PCFP	XGB	{'learning_rate': 0.01, 'max_depth': 10, 'n_estimators': 1500}
CYP3A4	MACCS+ECFP6	GBM	{'learning_rate': 0.01, 'max_depth': 10, 'max_features': 'log2', 'n_estimators': 3000}
	MACCS+ECFP6	LGBM	{'learning_rate': 0.01, 'n_estimators': 2000}
	MACCS+ECFP6	RF	{'max_features': 'auto', 'n_estimators': 2500}
	MACCS+ECFP6	XGB	{'learning_rate': 0.01, 'max_depth': 15, 'n_estimators': 3000}
	MACCS+FCFP4	GBM	{'learning_rate': 0.01, 'max_depth': 10, 'max_features': 'sqrt', 'n_estimators': 2500}
	MACCS+FCFP4	LGBM	{'learning_rate': 0.01, 'n_estimators': 2000}
	MACCS+FCFP4	RF	{'max_features': 'auto', 'n_estimators': 1500}
	**MACCS+FCFP4**	**XGB**	**{'learning_rate': 0.01, 'max_depth': 10, 'n_estimators': 2000}**
	MACCS+PCFP	GBM	{'learning_rate': 0.01, 'max_depth': 10, 'max_features': 'sqrt', 'n_estimators': 1500}
	MACCS+PCFP	LGBM	{'learning_rate': 0.01, 'n_estimators': 1500}
	MACCS+PCFP	RF	{'max_features': 'auto', 'n_estimators': 3000}
	MACCS+PCFP	XGB	{'learning_rate': 0.01, 'max_depth': 10, 'n_estimators': 1500}
CYP2C9	MACCS+ECFP6	GBM	{'learning_rate': 0.01, 'max_depth': 10, 'max_features': 'log2', 'n_estimators': 3000}
	MACCS+ECFP6	LGBM	{'learning_rate': 0.01, 'n_estimators': 3000}
	MACCS+ECFP6	RF	{'max_features': 'log2', 'n_estimators': 3000}
	MACCS+ECFP6	XGB	{'learning_rate': 0.01, 'max_depth': 15, 'n_estimators': 1500}
	MACCS+FCFP4	GBM	{'learning_rate': 0.01, 'max_depth': 10, 'max_features': 'log2', 'n_estimators': 3000}
	MACCS+FCFP4	LGBM	{'learning_rate': 0.01, 'n_estimators': 2500}
	MACCS+FCFP4	RF	{'max_features': 'auto', 'n_estimators': 3000}
	MACCS+FCFP4	XGB	{'learning_rate': 0.01, 'max_depth': 10, 'n_estimators': 2000}
	MACCS+PCFP	GBM	{'learning_rate': 0.01, 'max_depth': 10, 'max_features': 'sqrt', 'n_estimators': 1500}
	**MACCS+PCFP**	**LGBM**	**{'learning_rate': 0.01, 'n_estimators': 2000}**
	MACCS+PCFP	RF	{'max_features': 'auto', 'n_estimators': 3000}
	MACCS+PCFP	XGB	{'learning_rate': 0.01, 'max_depth': 10, 'n_estimators': 1500}

Table S8. Optimal Hyperparameters of QSAR Models for ADME/T Classification Tasks (continue)

Task	Descriptor	Method	Optimal Hyperarameters
hERG	MACCS+ECFP6	GBM	{'learning_rate': 0.05, 'max_depth': 15, 'max_features': 'log2', 'n_estimators': 1500}
	MACCS+ECFP6	LGBM	{'learning_rate': 0.01, 'n_estimators': 1000}
	MACCS+ECFP6	RF	{'max_features': 'log2', 'n_estimators': 2500}
	MACCS+ECFP6	XGB	{'learning_rate': 0.01, 'max_depth': 10, 'n_estimators': 1000}
	MACCS+FCFP4	GBM	{'learning_rate': 0.05, 'max_depth': 15, 'max_features': 'sqrt', 'n_estimators': 500}
	MACCS+FCFP4	LGBM	{'learning_rate': 0.05, 'n_estimators': 50}
	**MACCS+FCFP4**	**RF**	**{'max_features': 'auto', 'n_estimators': 1500}**
	MACCS+FCFP4	XGB	{'learning_rate': 0.05, 'max_depth': 10, 'n_estimators': 100}
	MACCS+PCFP	GBM	{'learning_rate': 0.05, 'max_depth': 10, 'max_features': 'sqrt', 'n_estimators': 2000}
	MACCS+PCFP	LGBM	{'learning_rate': 0.05, 'n_estimators': 3000}
	MACCS+PCFP	RF	{'max_features': 'auto', 'n_estimators': 2500}
	MACCS+PCFP	XGB	{'learning_rate': 0.05, 'max_depth': 15, 'n_estimators': 50}
AMES	MACCS+ECFP6	GBM	{'learning_rate': 0.01, 'max_depth': 10, 'max_features': 'sqrt', 'n_estimators': 1000}
	MACCS+ECFP6	LGBM	{'learning_rate': 0.01, 'n_estimators': 500}
	MACCS+ECFP6	RF	{'max_features': 'auto', 'n_estimators': 3000}
	MACCS+ECFP6	XGB	{'learning_rate': 0.01, 'max_depth': 15, 'n_estimators': 500}
	MACCS+FCFP4	GBM	{'learning_rate': 0.01, 'max_depth': 10, 'max_features': 'sqrt', 'n_estimators': 500}
	MACCS+FCFP4	LGBM	{'learning_rate': 0.01, 'n_estimators': 500}
	**MACCS+FCFP4**	**RF**	**{'max_features': 'auto', 'n_estimators': 1000}**
	MACCS+FCFP4	XGB	{'learning_rate': 0.01, 'max_depth': 15, 'n_estimators': 500}
	MACCS+PCFP	GBM	{'learning_rate': 0.01, 'max_depth': 10, 'max_features': 'sqrt', 'n_estimators': 500}
	MACCS+PCFP	LGBM	{'learning_rate': 0.05, 'n_estimators': 100}
	MACCS+PCFP	RF	{'max_features': 'auto', 'n_estimators': 2000}
	MACCS+PCFP	XGB	{'learning_rate': 0.01, 'max_depth': 10, 'n_estimators': 500}
DILI	MACCS+ECFP6	GBM	{'learning_rate': 0.05, 'max_depth': 10, 'max_features': 'sqrt', 'n_estimators': 500}
	MACCS+ECFP6	LGBM	{'learning_rate': 0.01, 'n_estimators': 500}
	MACCS+ECFP6	RF	{'max_features': 'auto', 'n_estimators': 2000}
	MACCS+ECFP6	XGB	{'learning_rate': 0.01, 'max_depth': 10, 'n_estimators': 500}
	MACCS+FCFP4	GBM	{'learning_rate': 0.01, 'max_depth': 15, 'max_features': 'log2', 'n_estimators': 50}
	MACCS+FCFP4	LGBM	{'learning_rate': 0.05, 'n_estimators': 100}
	MACCS+FCFP4	RF	{'max_features': 'log2', 'n_estimators': 1500}
	**MACCS+FCFP4**	**XGB**	**{'learning_rate': 0.05, 'max_depth': 15, 'n_estimators': 100}**
	MACCS+PCFP	GBM	{'learning_rate': 0.05, 'max_depth': 10, 'max_features': 'log2', 'n_estimators': 50}
	MACCS+PCFP	LGBM	{'learning_rate': 0.01, 'n_estimators': 1000}
	MACCS+PCFP	RF	{'max_features': 'auto', 'n_estimators': 2500}
	MACCS+PCFP	XGB	{'learning_rate': 0.01, 'max_depth': 10, 'n_estimators': 500}
